# Reversible photoswitching of the DNA-binding properties of styrylquinolizinium derivatives through photochromic [2 + 2] cycloaddition and cycloreversion

**DOI:** 10.3762/bjoc.16.13

**Published:** 2020-01-23

**Authors:** Sarah Kölsch, Heiko Ihmels, Jochen Mattay, Norbert Sewald, Brian O Patrick

**Affiliations:** 1 Department of Chemistry and Biology, Organic Chemistry II, University of Siegen, Adolf-Reichwein-Str. 2, D-57068 Siegen, Germany; 2 Department of Chemistry, Organic and Bioorganic Chemistry, Bielefeld University, PO Box 100121, D-33501 Bielefeld, Germany; 3 Department of Chemistry, Structural Chemistry Facility, The University of British Columbia, 2036 Main Mall, V6T 1Z1, Vancouver, BC, Canada

**Keywords:** azoniahetarenes, DNA ligands, photodimerization, photoswitches

## Abstract

It was demonstrated that styrylquinolizinium derivatives may be applied as photoswitchable DNA ligands. At lower ligand:DNA ratios (≤1.5), these compounds bind to duplex DNA by intercalation, with binding constants ranging from *K*_b_ = 4.1 × 10^4^ M to 2.6 × 10^5^ M (four examples), as shown by photometric and fluorimetric titrations as well as by CD and LD spectroscopic analyses. Upon irradiation at 450 nm, the methoxy-substituted styrylquinolizinium derivatives form the corresponding *syn* head-to-tail cyclobutanes in a selective [2 + 2] photocycloaddition, as revealed by X-ray diffraction analysis of the reaction products. These photodimers bind to DNA only weakly by outside-edge association, but they release the intercalating monomers upon irradiation at 315 nm in the presence of DNA. As a result, it is possible to switch between these two ligands and likewise between two different binding modes by irradiation with different excitation wavelengths.

## Introduction

The association of DNA-targeting drugs with nucleic acids [1–8] is considered one of the essential properties that determine their biological activity [9]. Specifically, a ligand may occupy particular binding sites of DNA or induce significant structural changes of the nucleic acid. In turn, both of these processes interfere with biologically relevant recognition processes between DNA and enzymes, e.g., topoisomerase [10]. Therefore, many potential lead structures of chemotherapeutic anticancer drugs exhibit DNA-binding properties [1–10]. Nevertheless, most DNA-binding ligands have an insufficient selectivity towards the targeted nucleic acid, and they also accumulate in healthy tissue, so that the chemotherapeutic treatment of tumors with DNA-binding drugs still suffers from severe side effects because of the intrinsic toxicity of the employed drugs [11–13]. As a result, there is an urgent need for DNA-targeting chemotherapeutic reagents that can be activated with an external stimulus only at the desired point of action. In this context, light offers several distinct advantages to switch on the activity of an otherwise inactive substrate (prodrug) because light is noninvasive, traceless, and easy to apply, and it enables local and temporal control [14]. To this end, photochromic systems appear to be highly attractive as a basis for photocontrollable substrates because they allow to switch the biological activity on and off due to the reversibility of the photoreaction [15]. Indeed, the application of light to induce and control bioactivity of pharmaceuticals or bio(macro)molecules has been convincingly demonstrated in the emerging field of photopharmacology [16–18]. Consequently, several attempts have also been made to develop photochromic DNA binders. Thus, it has been shown with spiropyran [19–21], stilbene [22–23], azobenzene [24–28], dithienylethene [29–32], chromene [33], and spirooxazine [34] derivatives that specifically modified photochromic ligands bind to DNA only with one of the components of the photochromic equilibrium. Moreover, these ligand–DNA interactions can be photochemically switched between the binding and nonbinding form. Interestingly, the photochromic systems applied in this context are almost exclusively photoinduced electrocyclization or *E*-to-*Z* isomerization reactions, whereas the well-established photochromic cycloaddition–cycloreversion equilibrium to establish photoswitchable DNA binders has so far been widely neglected. In fact, there is only one reported example for the use of the reversible photoinduced dimerization of stilbene derivatives as photoswitchable DNA ligand [35], and in this case, the structure of the photoproduct was not fully identified. Also, it has been shown that a DNA-binding azoniatetracene may be generated by photoinduced [4 + 4] cycloreversion. However, this system was not applied for photoinduced switching of binding properties [36]. Apparently, styryl-substituted aromatic derivatives could fill this gap because the [2 + 2] photocyclization reaction of stilbenes and derivatives thereof is a well-established reversible photoreaction [37–46], and styryl dyes, in particular cationic ones, were shown to be efficient DNA binders [47–58]. Nevertheless, the photochromic nature of DNA-binding styryl dyes has not been applied to use them as photoswitchable DNA binders. Although, there is one reported example that demonstrates the deactivation of a stilbene tyrosine kinase inhibitor by a [2 + 2] photocycloaddition [59].

As the quinolizinium ion has been established as a versatile platform for the development of DNA intercalators [60], we identified styryl-substituted quinolizinium derivatives as a promising basis for the search for photoswitchable DNA binders based on the photocycloaddition–photocycloreversion equilibrium. In fact, some selected styrylquinolizinium derivatives have already been shown to bind to DNA [61–67], however, their photocycloaddition reaction and the propensity of the corresponding photodimers to release the DNA-binding ligand have not been reported so far. Herein, we report on the photochemical and DNA-binding properties of the selected styrylquinolizinium derivatives **3a**–**d** and demonstrate their ability to operate as photoswitchable DNA ligands.

## Results and Discussion

### Synthesis

**Scheme 1:**
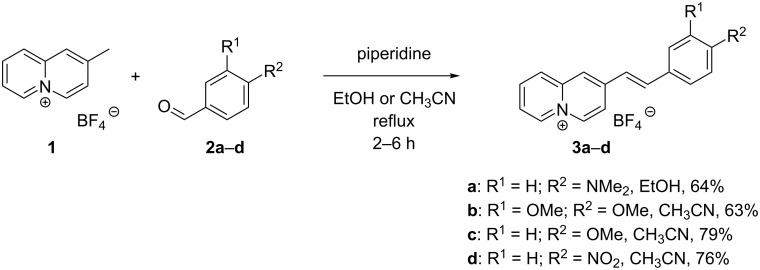
Synthesis of styrylquinolizinium derivatives **3a**–**d**.

2-Methylquinolizinium tetrafluoroborate (**1**) was synthesized according to published procedures [68]. The piperidine-catalyzed reaction of the latter with the benzaldehyde derivatives **2a**–**d** gave the 2-styrylquinolizinium derivatives **3a**–**d** in 63–79% yield (Scheme 1). The known products **3a** and **3c** were identified by comparison with literature data [69], and the new compounds **3b** and **3d** were fully characterized by NMR spectroscopy (^1^H, ^13^C, COSY, HSQC, and HMBC), elemental analyses, and mass spectrometry. In all cases, *E*-configuration of the alkene double bonds in **3a**–**d** was indicated by characteristic coupling constants of the alkene protons (^3^*J*_H–H_ = 16 Hz) [70].

### Absorption and emission properties

**Figure 1:**
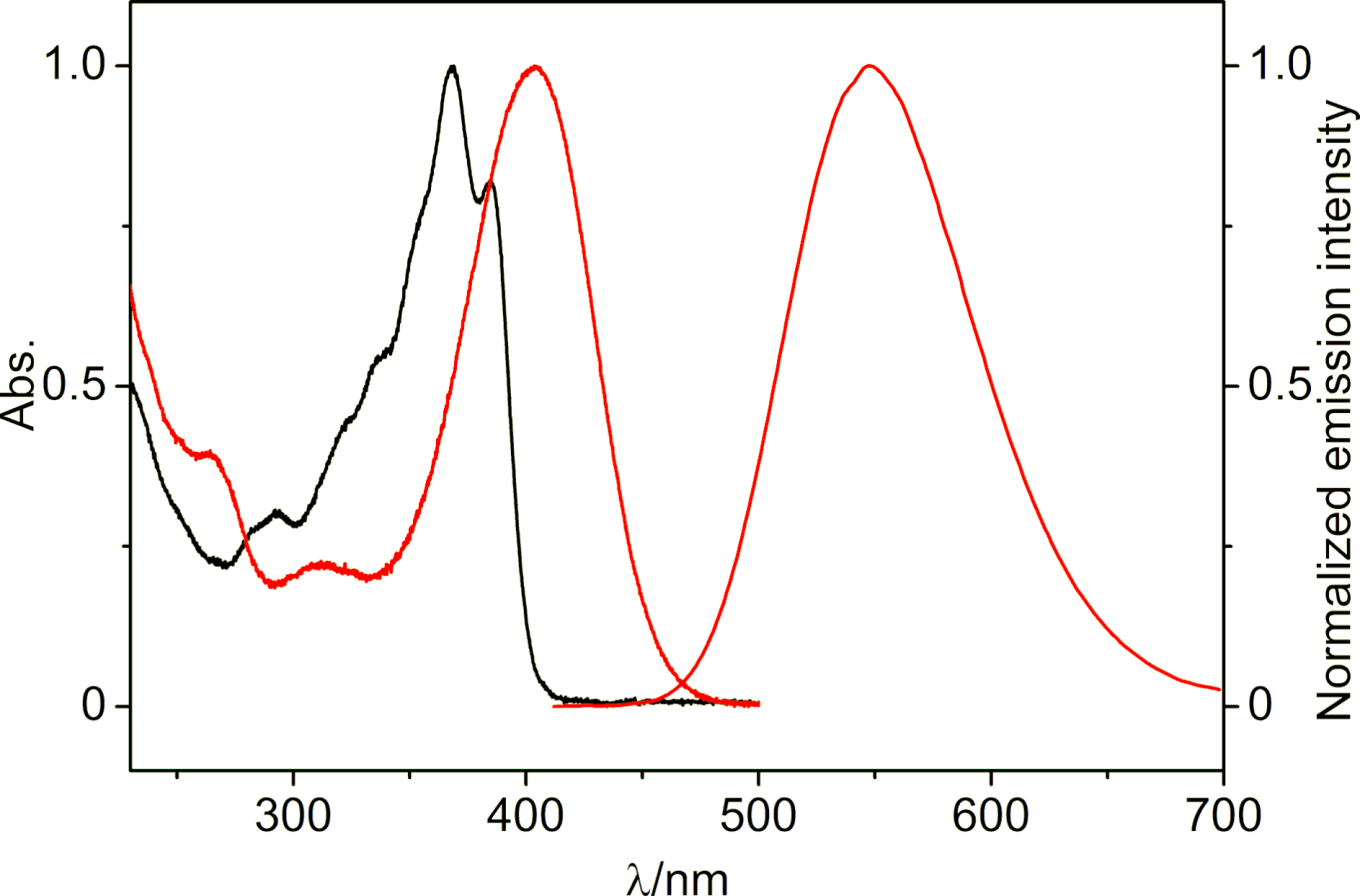
Absorption spectra and normalized emission spectrum (Abs. = 0.10, **3b**: λ_ex_ = 394 nm) of derivatives **3b** (red) and **3d** (black) in MeCN.

The photophysical properties of the styrylquinolizinium derivatives **3a** and **3c** have already been reported [69], while the ones of **3b** and **3d** were determined in this work (Table 1 and Figure 1). In acetonitrile, the derivatives **3b** and **3d** exhibited long-wavelength absorption maxima at λ_abs_ = 404 nm and 368 nm, with emission bands at λ_fl_ = 548 nm and 419 nm. Derivative **3d** was essentially nonfluorescent (Φ_fl_ < 0.01 in MeCN), whereas compound **3b** (Φ_fl_ = 0.17 in MeCN) had the largest fluorescence quantum yield in comparison to the derivatives **3a** (Φ_fl_ = 0.02 in MeCN) and **3c** (Φ_fl_ = 0.04 in MeCN). In aqueous solution, the compounds exhibited long-wavelength absorption maxima at 434 nm (**3a**), 389 nm (**3b**), 384 nm (**3c**), and 371 nm (**3d**) as well as weak emission bands at 630 nm (**3a**), 538 nm (**3b**), and 507 nm (**3c**). In contrast, the emission intensity of **3d** was too low to identify a maximum, as usually observed with nitro-substituted fluorophores. Unfortunately, the emission quantum yields of **3a**–**c** could not be determined in water because of the compounds’ tendency to dimerize even at very low concentrations (see below). Overall, the absorption and emission data revealed a significantly less pronounced donor–acceptor interplay in the methoxy-substituted derivatives **3b** and **3c** as compared to the strong donor–acceptor system **3a**, as clearly indicated by the blue-shifted absorption and emission bands of **3b** and **3c**. Consequently, the absorption bands of the electron acceptor-substituted derivative **3d** were shifted to even shorter wavelengths.

**Table 1:** Absorption and emission data for styrylquinolizinium derivatives **3a**–**d** in MeCN and water.

	MeCN				H_2_O	
			
	λ_abs_/nm^a^	λ_fl_/nm^b^	Φ_fl_^c^		λ_abs_/nm^a^	λ_fl_/nm^b^

**3a**^d^	474	643	0.02		434	630
**3b**	404	548	0.17^e^		389	538
**3c**^d^	392	517	0.04		384	507
**3d**	368	419	<0.01^f^		371	–^g^

^a^Long-wavelength absorption maximum, *c*(**3b**/**3d**) = 20 µM. ^b^Fluorescence maximum, λ_ex_ = 394 nm (**3b**) and 370 nm (**3d**). ^c^Emission quantum yield, determined with Abs = 0.10 at λ_ex_, estimated error of Φ_fl_: ±10%. ^d^Taken from [71]. ^e^Relative to coumarin 152 (Φ_fl_ = 0.28) [71]. ^f^Relative to coumarin 1 (Φ_fl_ = 1.00) [71]. ^g^Too weak to be determined.

### DNA-binding properties

**Figure 2:**
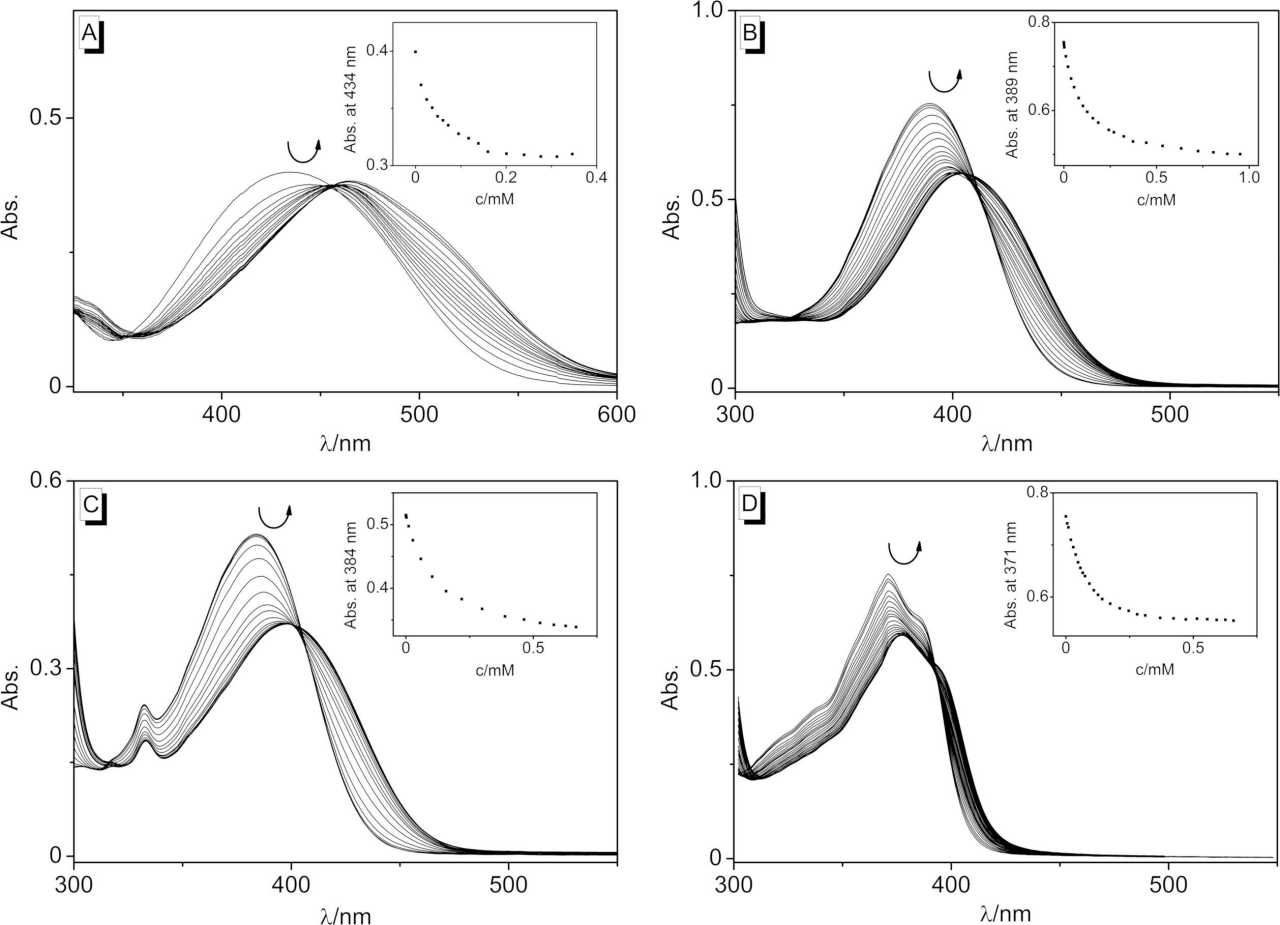
Spectrophotometric titration upon the addition of ct DNA to the styrylquinolizinium derivatives **3a** (A), **3b** (B), **3c** (C), and **3d** (D) in BPE [*c*_L_ = 20 µM, *c*_DNA_ = 1.45 mM (A–C), *c*_DNA_ = 2.45 mM (D), *c*_DNA_ in base pairs]. The insets show the plots of absorption vs DNA concentration. The arrows indicate the changes of absorption upon the addition of ct DNA.

The DNA-binding properties of the 2-styrylquinolizinium derivatives **3a**–**d** were investigated by spectrometric titrations of calf thymus DNA (ct DNA) to **3a**–**d** in a phosphate buffered solution at pH 7.0 (Figure 2). During the photometric titrations, the initial absorption maxima continuously decreased and new, bathochromically shifted absorption maxima arose at 464 nm (**3a**), 404 nm (**3b**), 399 nm (**3c**), and 378 nm (**3d**), respectively (Figure 2), which clearly indicated the association of these ligands with the nucleic acid [72]. In all cases, isosbestic points developed at the beginning of the titration and eventually became indistinct, which already indicated different binding modes at particular stages of the titration.

The data from the photometric titrations are presented as binding isotherms, and fitting of the experimental data to an established theoretical model [73] gave the corresponding binding constants *K*_b_ (cf. Supporting Information File 1). Thus, the largest binding constant was determined for the dimethylamino-substituted styrylquinolizinium derivative **3a** (*K*_b_ = 2.6 ± 0.1 × 10^5^ M). The nitro-substituted derivative **3d** had a slightly lower affinity with *K*_b_ = 8.2 ± 0.2 × 10^4^ M, and the methoxy-substituted derivatives had the lowest binding constants of *K*_b_ = 4.8 ± 0.1 × 10^4^ M (**3b**) and 4.1 ± 0.1 × 10^4^ M (**3c**). Overall, these binding affinities resembled the ones of known DNA-intercalating benzoquinolizinium derivatives [60].

**Figure 3:**
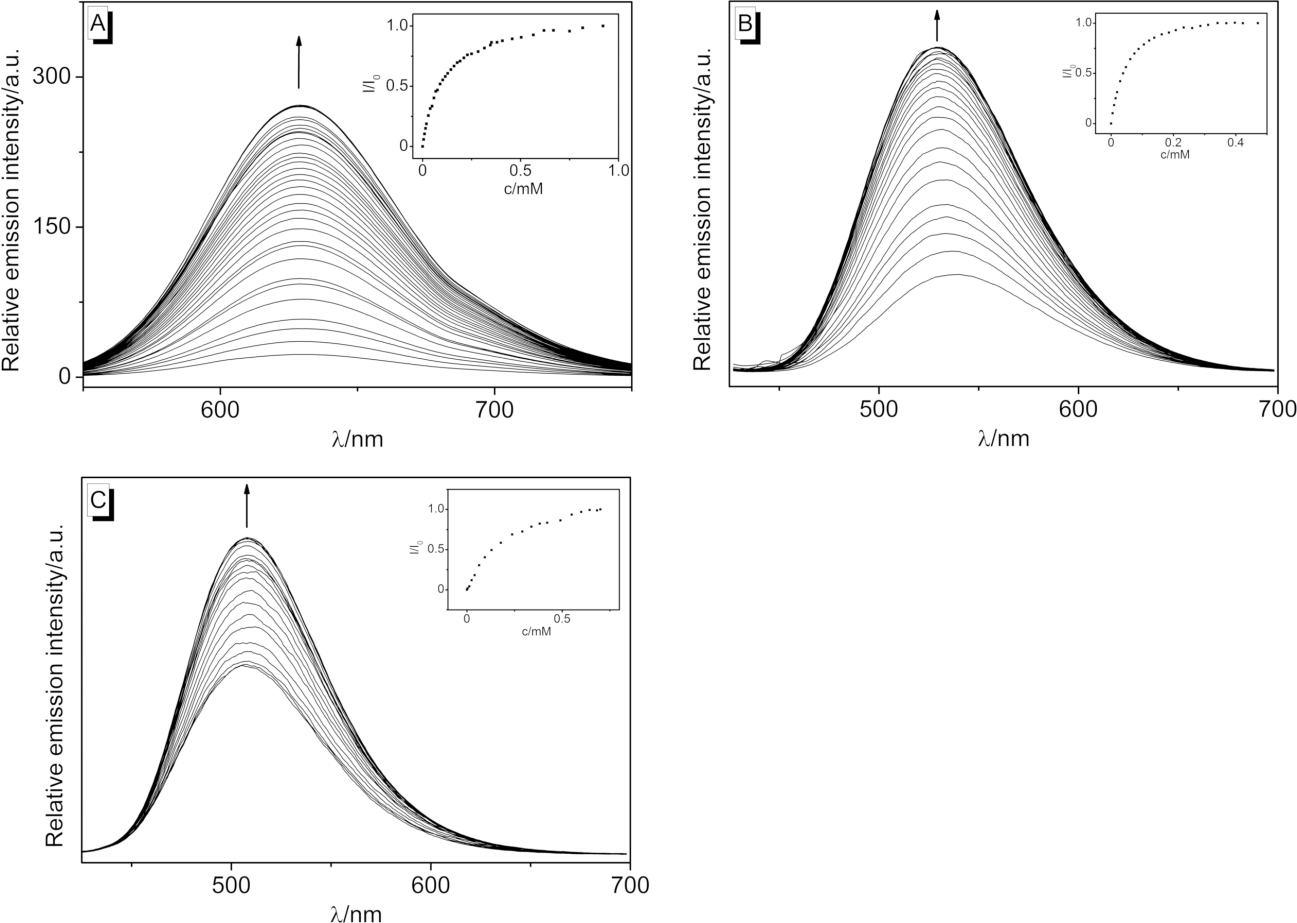
Spectrofluorimetric titration upon the addition of ct DNA to the styrylquinolizinium derivatives **3a** (A), **3b** (B) and **3c** (C) in BPE buffer [*c*_L_ = 5 µM (A, C), *c*_L_ = 1 µM (B), *c*_DNA_ = 1.45 mM, *c*_DNA_ in base pairs]. The insets show the plots of relative emission intensity vs DNA concentration. The arrows indicate the changes of emission intensity upon the addition of ct DNA.

In addition, the changes of the emission properties upon the addition of ct DNA to 2-styrylquinolizinium derivatives **3a**–**d** were determined in fluorimetric titrations (Figure 3). The intensity of the rather weak emission bands of **3a**, **3b**, and **3c** increased significantly upon the addition of DNA. In the case of derivative **3b**, a blue-shift of the emission maximum by 10 nm was also observed. Notably, compound **3a** had the weakest emission intensity, i.e., it was essentially nonfluorescent in aqueous solution, but when it was bound to DNA, it showed a strong light-up effect of the emission with a factor of *I*/*I*_0_ = 44. For compounds **3b** and **3c**, significantly smaller light-up factors of *I*/*I*_0_ = 3.3 and 1.6, respectively, were observed. In contrast, the very low emission intensity of **3d** did not change upon the addition of ct DNA. The fluorescence light-up effects of the ligands **3a**–**c** upon association with DNA resembled the ones observed for other styryl-substituted quinolizinium derivatives [62–67]. Accordingly, the emission enhancement most likely resulted from the accommodation of the ligand in the constrained binding site of the DNA, which led to a restricted conformational flexibility. As a result, conformational changes of the styryl substituent in the excited state that lead to radiationless deactivation in solution were significantly suppressed within the binding site so that emission became competitive.

**Figure 4:**
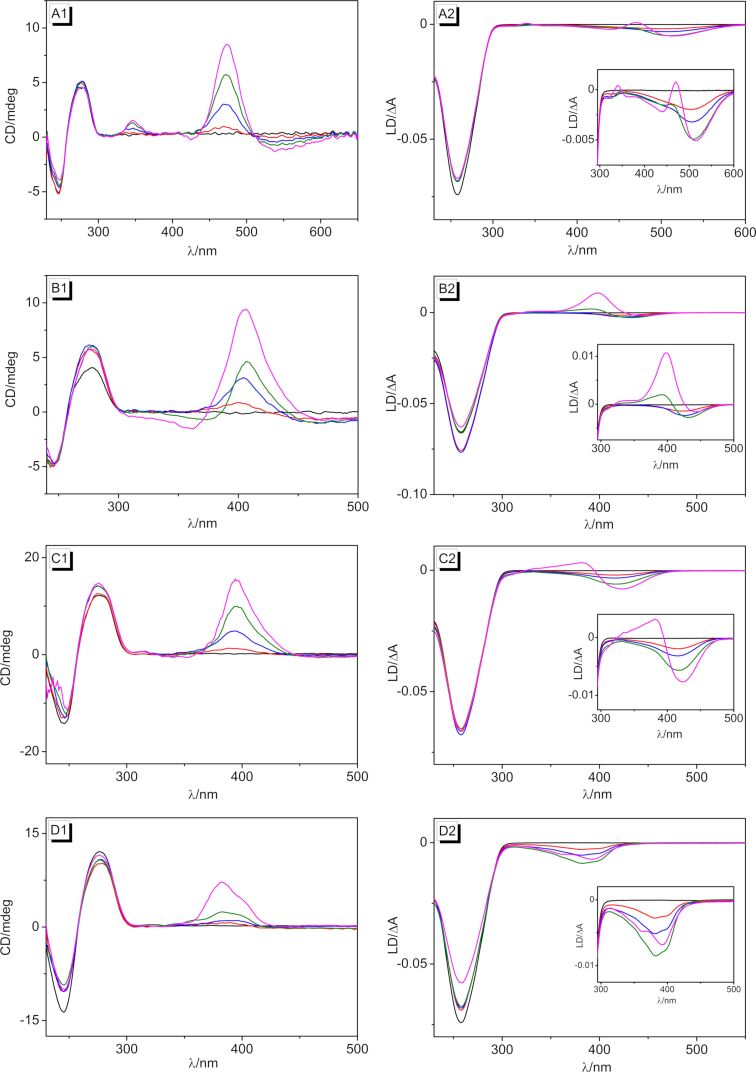
CD and LD spectra of the styryl derivatives **3a** (A), **3b** (B), **3c** (C), and **3d** (D) with ct DNA in BPE buffer [*c*_ct DNA_ = 20 µM (A1, B1), *c*_ct DNA_ = 50 µM (C1, D1), and *c*_ct DNA_ = 500 µM (A2–D2), with *LDR* = 0 (black), 0.5 (red), 1.0 (blue), 1.5 (green), and 2.0 (magenta), *c*_ct DNA_ in base pairs).

The DNA-binding properties of the ligands **3a**–**d** were further investigated by circular dichroism (CD) and flow linear dichroism (LD) spectroscopy [74–75] in phosphate buffer at different ligand-to-DNA ratios (LDR). The mixtures of compounds **3a**–**d** with ct DNA showed clear induced circular dichroism (ICD) and LD bands in the absorption region of the ligands that further confirmed the binding of the ligands (Figure 4). In all cases, a positive ICD signal developed, and with increasing LDR, the characteristic CD bands of duplex DNA at 254 nm and 277 nm [76] increased slightly. Ligand **3a** exhibited a strong positive and a weak negative ICD signal at 473 nm and 583 nm, respectively, in the presence of DNA, along with a weaker positive signal at 346 nm (Figure 4A1). For LD spectroscopic analysis, the DNA molecules were oriented in a hydrodynamic field of a rotating couette (flow linear dichroism). The corresponding LD spectra were the result of the differential absorption of linearly polarized light, which was polarized parallel and perpendicular to a reference axis, respectively, thus indicating the orientation of the transition moment of the chromophores relative to the electric field vector of the light [75]. The LD spectrum of DNA-bound **3a** displayed a negative band in the absorption range of the ligand at small LDR (≤1.0) at 506 nm, whereas at higher values, a positive band developed, which led to a distorted bisignate band. In the case of ligands **3b** and **3c**, a similar development of LD bands was observed with increasing LDR, however, the effect was more pronounced with a strong positive LD signal at 397 nm (**3b**) and 382 nm (**3c**) at LDR = 0.5 (Figure 4B2 and Figure 4C2). Interestingly, the CD spectra of **3b** and **3c** did not resemble the ones of **3a**. Both ligands showed a clear positive ICD band at 400–407 nm (**3b**) and 382 nm (**3c**), but only in the case of **3b**, a weak blue-shifted ICD band also appeared at lower LDR (Figure 4B1 and Figure 4C1). Ligand **3d** exhibited positive ICD and negative LD signals at 382 nm upon binding to DNA (Figure 4D). Altogether, the CD and LD spectra of ligands **3a**–**c** at low LDR as well as the ones of **3d** in general showed the characteristic signatures of DNA intercalators. Namely, the negative LD bands of the bound ligands unambiguously revealed an intercalative mode [75–76], whereas the positive ICD bands indicated an essentially perpendicular alignment of the transition dipole moments of the ligands relative to the ones of the DNA base pairs [75–76]. Considering a dipole moment of the donor–acceptor systems **3a**–**c** along the long molecular axis, a binding mode in which the ligand is accommodated in the intercalation site with its long molecular axis perpendicular to the long axis of the binding site could be deduced. With increasing LDR, however, another binding mode became predominant for the ligands **3a**–**c**, as particularly indicated by the development of a positive LD band in the absorption range of the ligand that denoted groove binding [74–76]. It is proposed that with increasing ligand concentration, i.e., at larger LDR, the ligands tended to form aggregates, as commonly observed for donor–acceptor dyes, that stacked along the grooves of DNA.

### Photocycloaddition reactions

**Figure 5:**
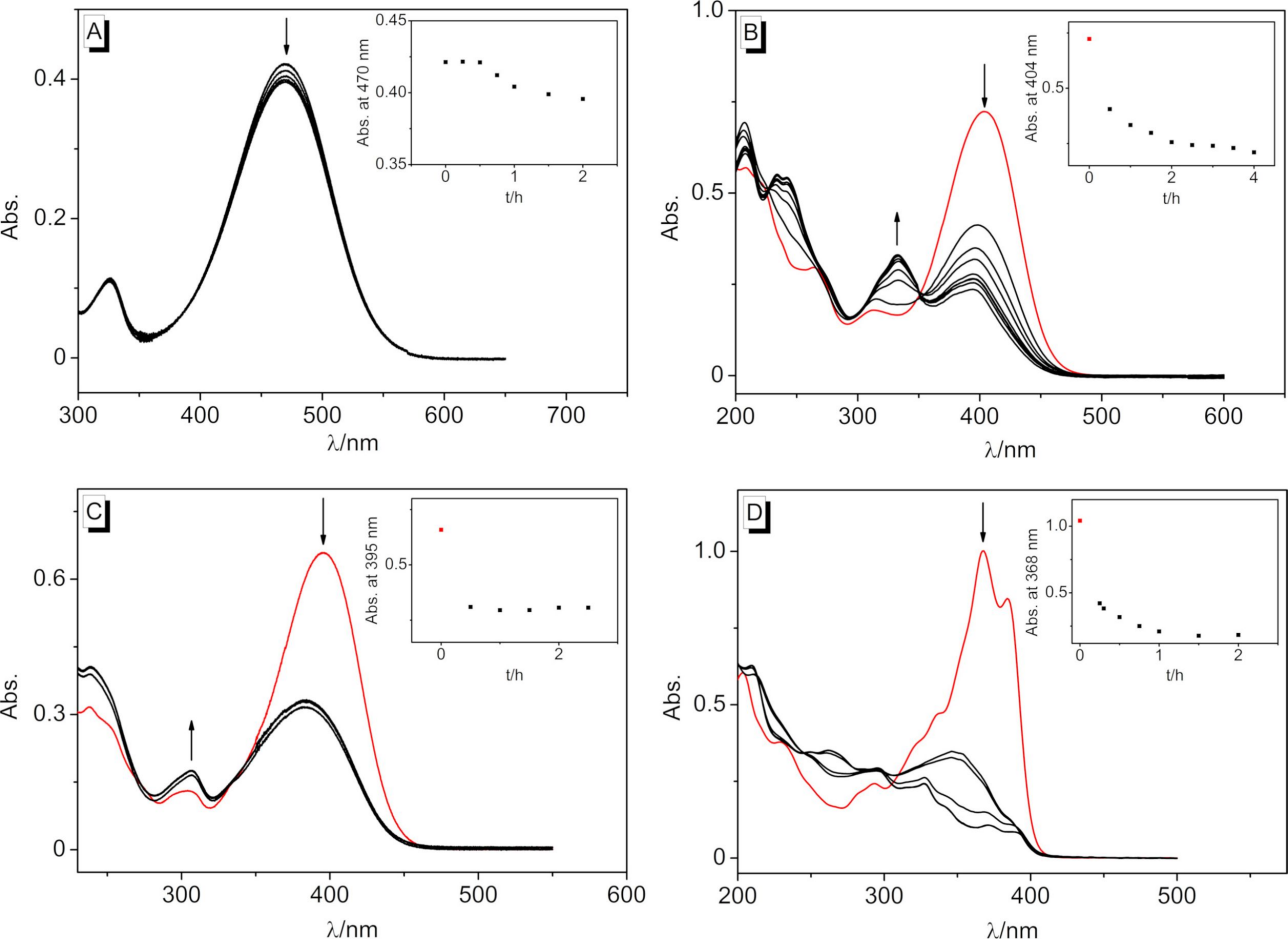
Spectrophotometric monitoring of the irradiation of styrylquinolizinium derivatives **3a** (A), **3b** (B), **3c** (C), and **3d** (D) in acetonitrile [*c*_L_ = 10 µM (A), *c*_L_ = 20 µM (B, C, D)]. The arrows indicate the changes of absorption upon irradiation.

The photochemical properties of the derivatives **3a**–**d** were investigated. Firstly, the substrates were irradiated in acetonitrile solution at 520–535 nm (**3a**), 420–470 nm (**3b** and **3c**), and >395 nm (**3d**), and the photoreaction was monitored photometrically. Notably, the amino-substituted derivative **3a** did not react under these conditions, as indicated by only marginal changes of the absorption spectrum (Figure 5A). Presumably, the strong donor–acceptor system in **3a** led to an intramolecular charge-transfer (ICT) state that did not lead to a subsequent photoreaction [77]. In contrast, the absorption bands of the substrates **3b**–**d** decreased relatively fast upon irradiation, but the maxima did not disappear completely (Figure 5B–D). Even after 4 h, compound **3b** exhibited a weak band at λ_abs_ = 404 nm, whereas the newly formed band at λ_abs_ = 332 nm did not increase further (Figure 5B). In this case, additional ^1^H NMR spectroscopic analysis showed that the derivatives **3c** and **3d** were initially converted to the *Z-*isomer by irradiation at λ = 450 nm or λ = 360 nm in acetonitrile, as indicated by the upfield shift of the signals of the alkene double bonds and the characteristic coupling constants of Z-configured protons (^3^*J_H–H_* = 12 Hz). Notably, the derivative **3c** did not react any further under these conditions (cf. Supporting Information File 1). However, it was observed that further irradiation of the nitro-substituted derivative **3d** furnished the dimer in acetonitrile, as shown by the development of the characteristic cyclobutane protons at 4.85–4.95 ppm. In contrast, the NMR-spectroscopic analysis in D_2_O showed that the derivative **3b** gave the corresponding cycloaddition product much faster, i.e., within a few minutes under these conditions, and the formation of the corresponding *Z*-isomer proceeded only to a marginal extent.

**Figure 6:**
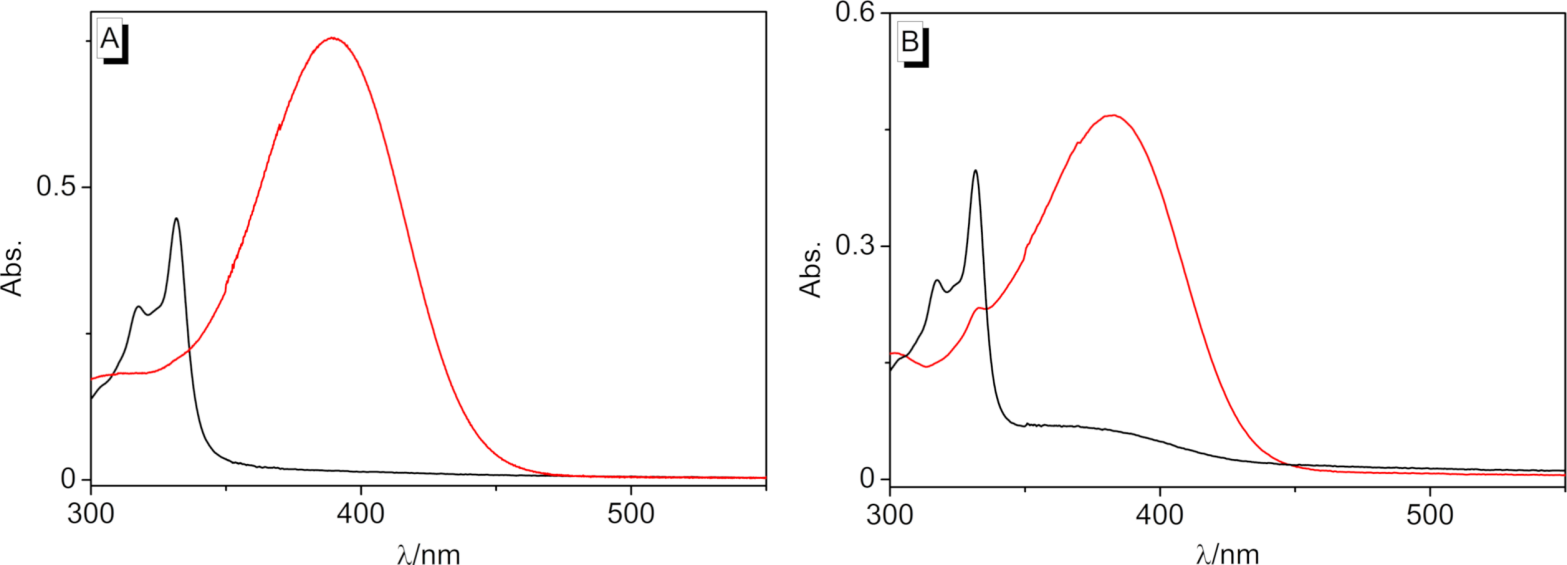
Absorption of the monomers (*c* = 20 µM, red) **3b** (A) and **3c** (B) and their dimers (black) **4b** and **4c** in H_2_O after 1.5 h and 4 h, respectively, at ca. 450 nm.

**Figure 7:**
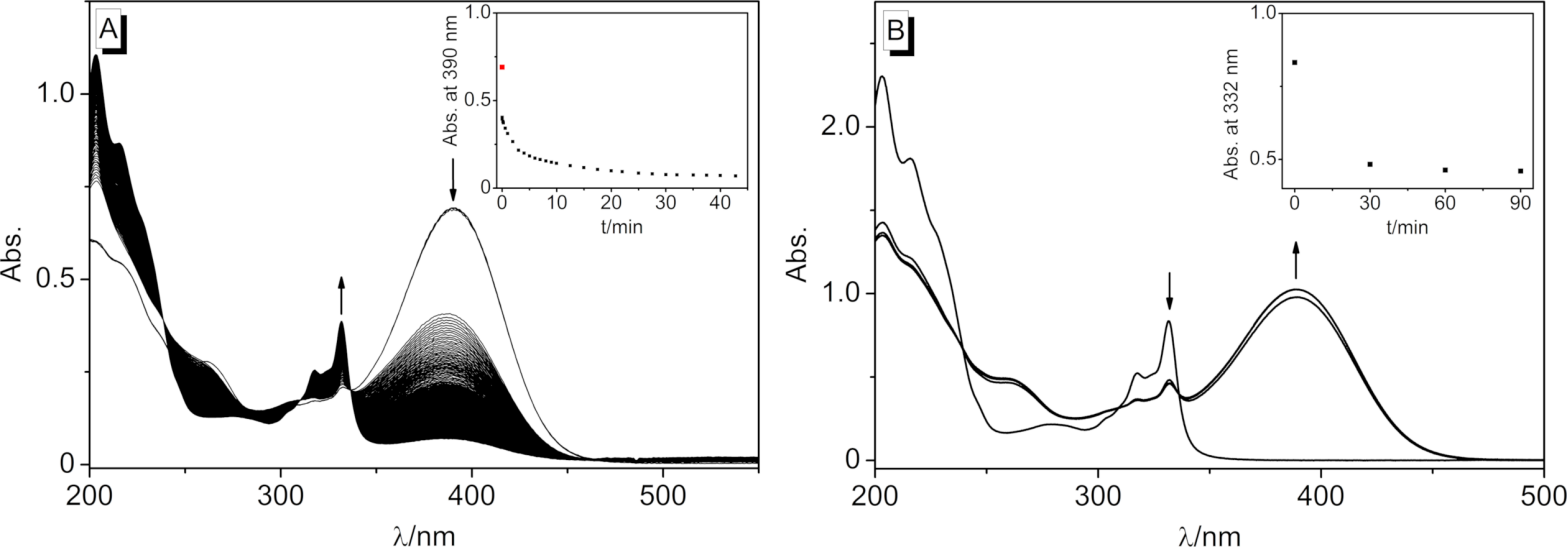
Photometric monitoring of the photoreaction of **3b** (*c* = 20 µM) to the dimer **4b** by irradiation at ca. 450 nm in H_2_O (A) and of the photoinduced cycloreversion of **4b** (*c* = 20 µM) to the monomer **3b** at 315 nm in H_2_O (B).

In aqueous solution, the substrates **3a**–**d** showed essentially the same photochemical behavior, however, with different reaction times and conversions. Thus, the photoreaction of derivative **3b** was complete after 90 min (Figure 6A), whereas the reaction of derivative **3c** took more than 5 h. The early stages of the photoreaction of substrate **3b** in water were monitored in short time intervals (1 s) to identify possible primary photoprocesses (Figure 7A). The initial maximum of the monomer **3b** decreased substantially by approximately half within a second, whereas further reaction was indicated by the appearance of the absorption maximum of **4b** at 317–331 nm. Notably, no additional intermediate absorption band appeared, and three isosbestic points developed at 239 nm, 310 nm, and 337 nm after the initial steps. These observations provided evidence that the phototransformation of the styrylquinolizinium species **3b** to its photodimer **4b** was a two-step process.

**Figure 8:**
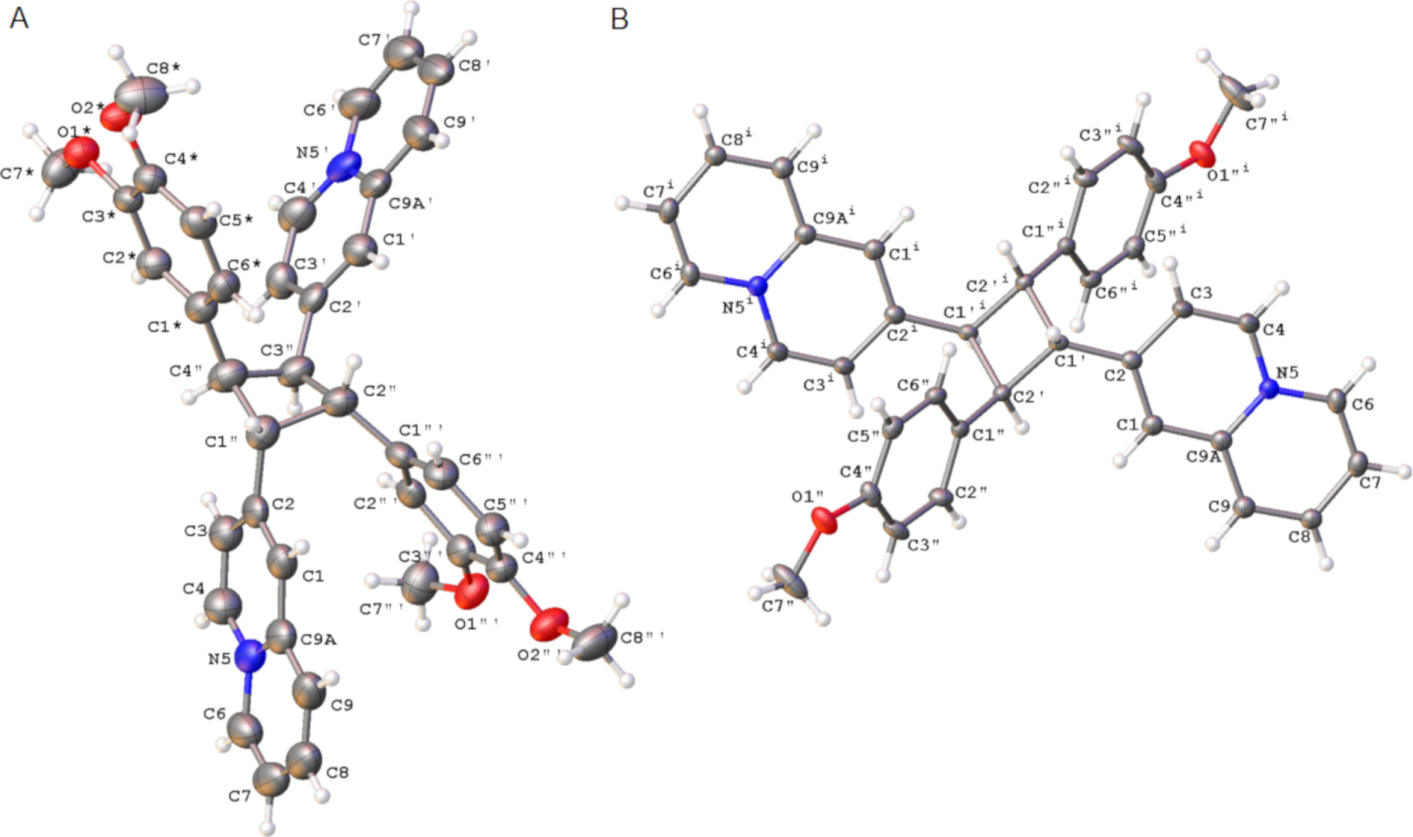
ORTEP drawings of cyclobutane derivatives **4b** (A) and **4c** (B) in the solid state (thermal ellipsoids indicate 50% probability). The tetrafluoroborate counterions were omitted for clarity.

Preparative-scale photoreactions were performed with the methoxy-substituted derivatives **3b** and **3c** because the photometric studies (see above) indicated reasonable reaction times. Unfortunately, it turned out that due to the low solubility of these derivatives in water, the concentrations required for a bimolecular reaction could not be accomplished. However, it is well known that [2 + 2] photodimerizations can also be performed in the solid state or with a thoroughly stirred suspension [37,43,78]. Therefore, suspensions of **3b** und **3c** in water were irradiated with an LED lamp at 450–470 nm to give the 2,2'-(2,4-diphenyl-1,3-cyclobutanediyl)bisquinolizinium **4b** and **4c** as photoproducts in quantitative yield. The products **4b** and **4c** were fully characterized by NMR spectroscopy (^1^H, ^13^C, COSY, HSQC, HMBC, and ROESY) and mass spectrometry, which revealed a cyclobutane structure, specifically by the appearance of the characteristic NMR signals of the cyclobutane at 4.89–5.00 ppm [42–46]. Unfortunately, detailed 2D NMR and spectroscopic analyses did not allow a conclusive assignment of the configuration of the products. Even in the ROESY NMR spectra, only unspecific correlations were detected. However, as both products could be obtained as single crystals after slow evaporation, their structure was determined by single crystal X-ray diffraction (XRD) analysis (Figure 8, cf. Supporting Information File 1). The cyclobutane **4b** crystallized from water in the monoclinic space group *P*2_1_/*n,* and the derivative **4c** crystallized from water in the triclinic space group 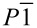. Both XRD analyses clearly showed that both cyclobutane products were formed as *rctt* configured dimers **4b** and **4c** (Figure 8).

**Scheme 2:**
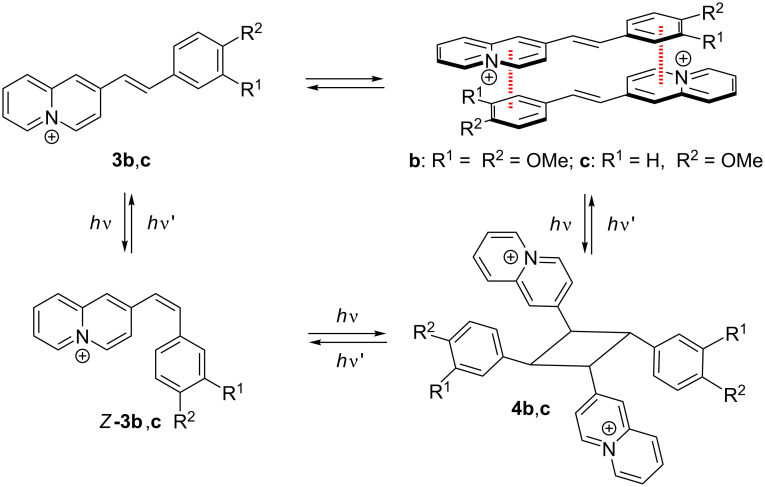
Possible pathways for the selective photodimerization of styrylquinolizinium derivatives **3b** and **3c**.

The products **4b** and **4c** may have formed by a *syn* head-to-tail dimerization of the *E*-configured substrate **3a** and **3b** or by an *anti* head-to-tail photodimerization of the initially formed *Z-*isomers *Z-***3a** and *Z-***3b**, with both processes generally being possible starting from **3b** and **3c** (Scheme 2). On the one hand, the photometric monitoring as well as the ^1^H NMR spectroscopic studies of the photoreaction of **3b** indicated a preceding *E*-to-*Z* isomerization (cf. Supporting Information File 1) that may have been followed by a [2 + 2] photodimerization (Scheme 2). However, it is difficult to explain why the photocycloaddition of the *Z*-isomers *Z-***3b** and *Z-***3c** led exclusively to the dimer, because such a selectivity has not been reported so far for (*Z)*-stilbenes. On the other hand, the reaction was performed using suspensions, so that the reaction may also have taken place with undissolved solid in which the *E*-to-*Z* isomerization was most likely suppressed due to the restricted space in the confined medium. Thus, the selective formation of the dimers **4b** and **4c** is reminiscent of the high stereoselectivity observed for [2 + 2] photodimerizations in organized media or in the solid state [37–41].

Considering the pronounced donor–acceptor interplay in **3b** and **3c** and the resulting strong dipole moment, it may be proposed that these compounds form dimeric aggregates in the solid state and even in solution through dipole–dipole interactions and directional π stacking (Scheme 2), as observed, for example, with donor-substituted benzoquinolizinium derivatives [79] or donor-substituted styrylpyridinium derivatives [80–82]. Hence, an ideal overlap of the π systems and antiparallel alignment of dipole moments is realized in a *syn* head-to-tail complex where irradiation would lead directly to the photodimers **4b** and **4c** in a topochemical reaction (Scheme 2).

Notably, the cyclobutane derivatives **4b** and **4c** were not persistent in solution for extended periods of time. As already shown for several cyclobutane derivatives, these compounds tend to isomerize to the corresponding *rttt* isomers [83–86].

With derivative **4b** as a representative example, it was demonstrated that the photodimers can be transformed back to the monomers. Thus, upon irradiation of cyclobutane **4b** at 315 nm in H_2_O, the monomer **3b** formed, as indicated by the development of its characteristic absorption band (Figure 7B). After 30 min, the reaction was almost complete, however, dimer **4b** still remained in solution in the photostationary state.

### Interactions of the photodimer **4b** with DNA

**Figure 9:**
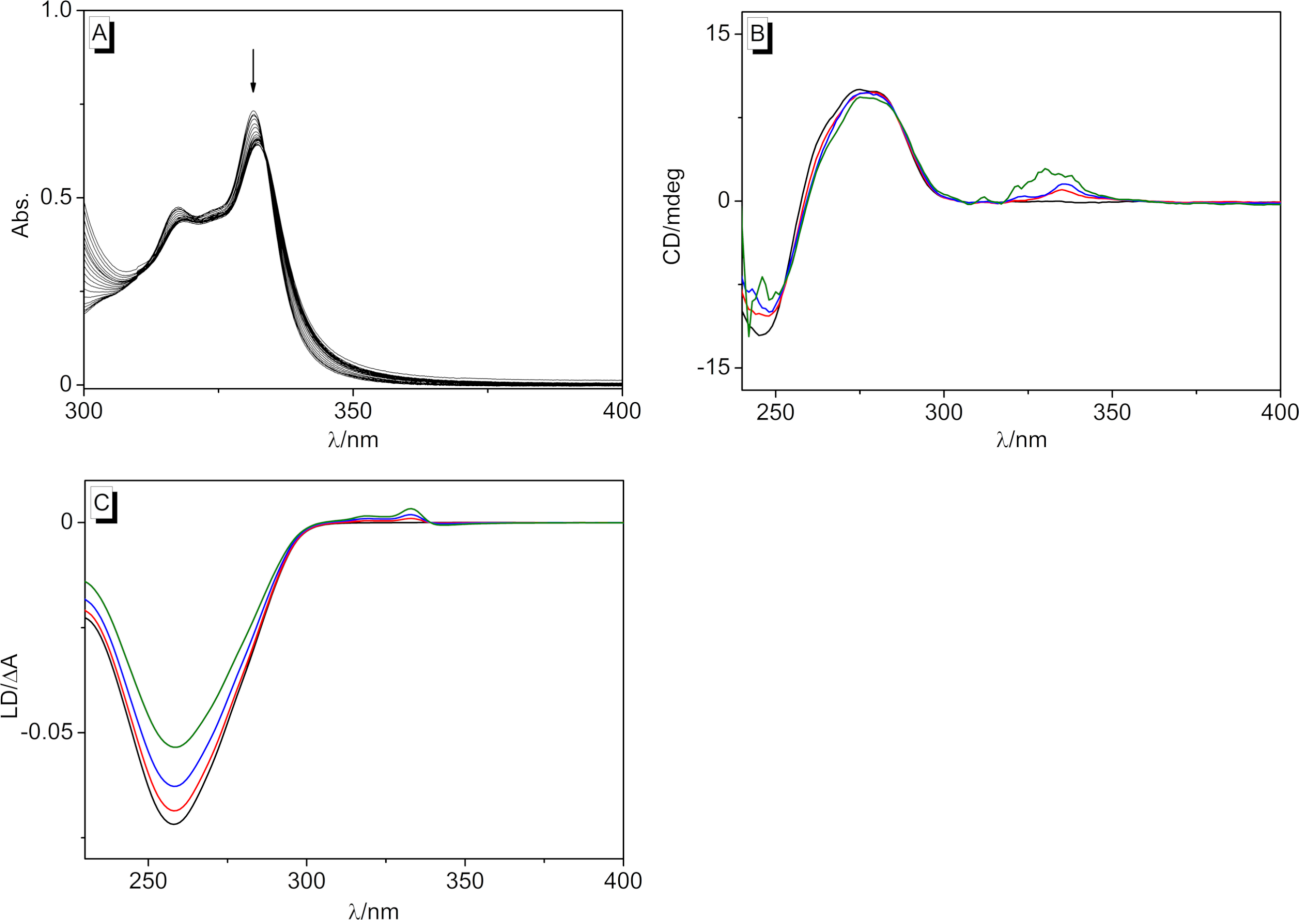
A) Spectrophotometric titration of ct DNA to dimer **4b** in BPE buffer (*c*_L_ = 20 µM, *c*_ct DNA_ = 1.45 mM, *c*_ct DNA_ in base pairs). The arrow indicates the changes of absorption upon the addition of ct DNA. B) CD spectra of the dimer **4b** with ct DNA (50 µM) in BPE buffer with LDR = 0 (black), 0.5 (red), 1.0 (blue), and 2.0 (green). C) LD spectra of dimer **4b** with ct DNA (c = 500 µM) in BPE buffer with LDR = 0 (black), 0.04 (red), 0.08 (blue), and 2.0 (green).

The interactions of dimer **4b** with DNA were investigated by photometric titrations as well as by CD and LD spectroscopy (Figure 9). Upon the addition of ct DNA to compound **4b** in buffered solution, the absorption maximum decreased slightly, but apart from a broadening of the band at the long-wavelength tail, the overall shape of the spectrum did not change (Figure 9). Furthermore, only a small positive ICD band in the absorption region of ligand **4b** appeared at 300–350 nm that developed into a significantly broader band with increasing LDR. At the same time, the signal of the DNA did not change in the presence of the ligand. Additionally, the LD experiment showed a small positive signal at 300–350 nm, and the negative band of the ct DNA at 254 nm decreased. These spectroscopic data indicated a very weak interaction of the substrate **4b** with DNA, and the band broadening in the absorption region already confirmed an aggregation of the molecules along the DNA backbone at very high ligand concentrations. Nevertheless, the CD and LD spectroscopic data revealed at least some specific binding interactions of **4b** with DNA that caused a distinct orientation of the aromatic units relative to the host DNA. In particular, the weak positive LD band indicated an alignment of the aromatic units along the DNA grooves. In addition, the close vicinity of the quinolizinium substituents to the DNA helix was further confirmed by the CD band in the absorption region of the quinolizinium moiety, as it resulted from the coupling of its transition dipole moment with the ones of the DNA bases. Overall, these data revealed a loose binding of the cyclobutane derivative **4b** to DNA through outside-edge binding of the ligand that enabled the association of one or two aromatic units in the DNA grooves.

### Photoswitching of the DNA binding properties

**Figure 10:**
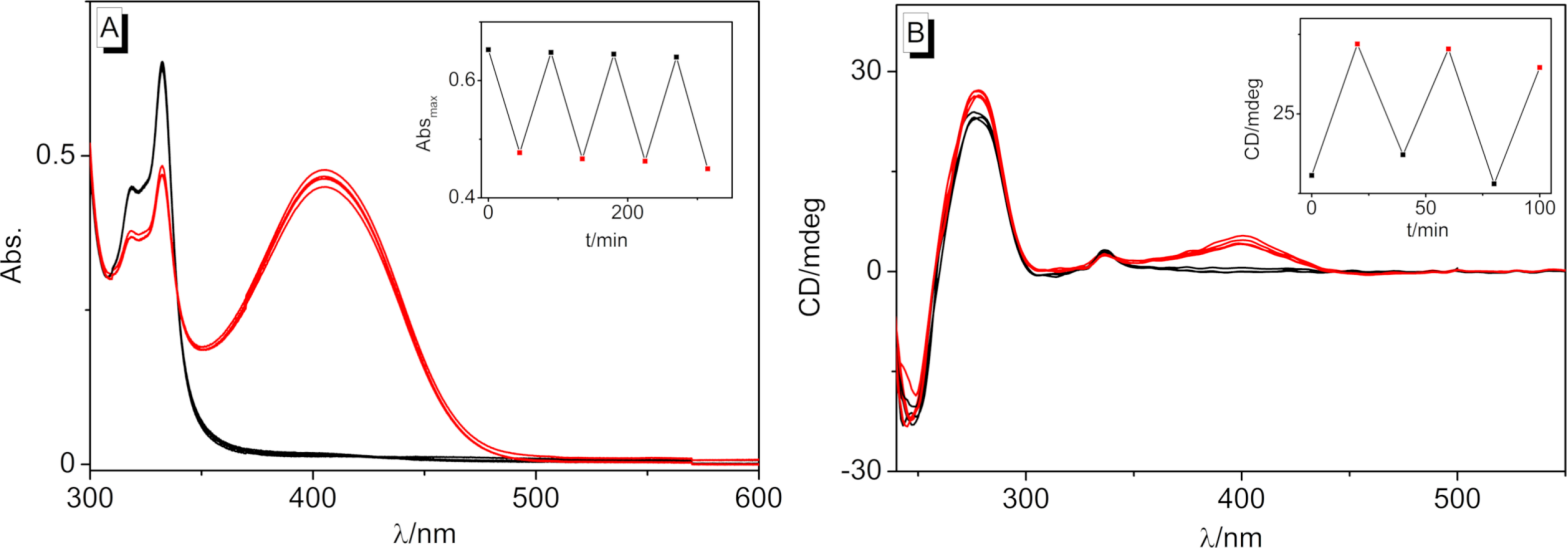
A) Photometric and B) CD spectroscopic monitoring of the photoinduced switching (**4b**: λ_ex_ = 315 nm, **3b**: λ_ex_ = 450 nm) between **4b** (*c* = 20 µM, black) and **3b** (red) in the presence of ct DNA (*c* = 20 µM) in BPE buffer. DNA concentration in base pairs.

Finally, it was tested whether the DNA-binding quinolizinium derivative **3b** could be released photochemically from cyclobutane **4b** in the presence of DNA. For that purpose, a mixture of the photodimer and DNA was irradiated at 315 nm using an LED, and the reaction was monitored by absorption and CD spectroscopy (Figure 10). In the course of the photoreaction, the formation of **3b** was indicated by the emergence of its characteristic long-wavelength absorption band, whose shape and shift matched its DNA-bound form. The association of the released monomer **3b** with DNA was also clearly demonstrated by the ICD band of the DNA-bound ligand. It should be noted, however, that the photoinduced conversion of the dimer was not complete, indicating a photostationary state. Noteworthy, irradiation of the bound ligand at ca. 450 nm using an LED regenerated the cyclobutane dimer, as shown unambiguously by the formation of the characteristic signature of its absorption and CD bands and by the disappearance of the monomer’s signals (Figure 10). Although the sequence of photocycloreversion and photoaddition could be performed four times, a slight but steady photobleaching or photodecomposition was observed. It should be noted that the DNA-bound ligand did not dimerize upon irradiation because within the intercalation site, it could not approach another quinolizinium molecule that was required for the photoreaction. Instead, the photodimerization most likely involved the free or loosely backbone-associated ligands that were in a dynamic equilibrium with the respective intercalator–DNA complexes, as shown for aryl stilbazonium ligands [35]. At the same time, the photoinduced cycloreversion may have taken place both with the free or DNA-bound dimer. Specifically, the dimer is only loosely bound to the DNA backbone so that the cycloreversion reaction does not experience steric constrains that may hinder the photoreaction. Furthermore, it has been demonstrated that the photoinduced cycloreversion of quinolizinium dimers is even enhanced in the presence of DNA [36].

## Conclusion

**Scheme 3:**
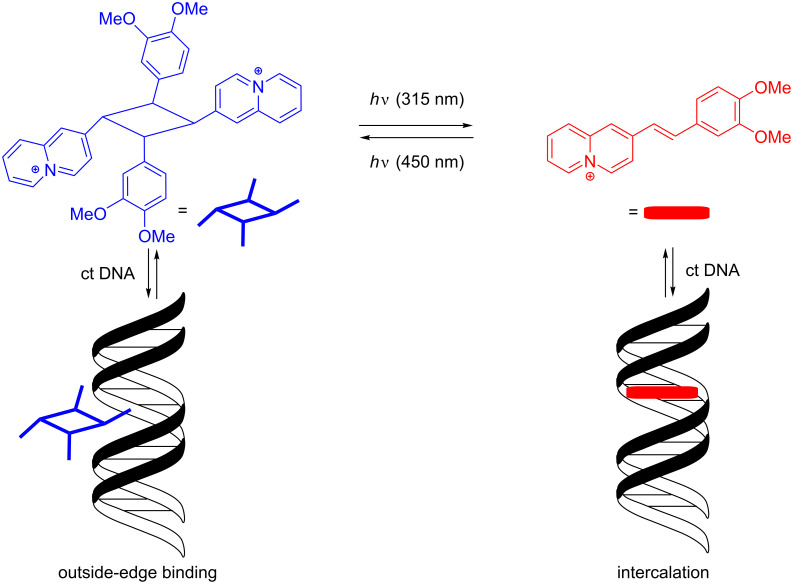
Photoinduced switching of the DNA binding properties of styrylquinolizinium compound **3b**.

In summary, we have shown that appropriately substituted styrylquinolizinium derivatives constitute a new class of photoswitchable DNA ligands. It was shown that these ligands bind to duplex DNA mainly by intercalation and that their *syn* head-to-tail photodimers, obtained by selective [2 + 2] photocycloaddition, bind to DNA only weakly by outside-edge association. Most notably, it was possible to switch between those two binding modes by irradiation with different excitation wavelengths (Scheme 3). Although the system still has to be improved with respect to photostability, it may be considered as a promising complementary approach toward the development of photoswitchable bioactive compounds.

## Supporting Information

File 1 Additional spectroscopic data, detailed experimental procedures, ^1^H NMR spectra, and crystallographic data.
