# Supplementary material for: Reversible photoswitching of the DNA-binding properties of styrylquinolizinium derivatives through photochromic [2 + 2] cycloaddition and cycloreversion
Source: Beilstein J Org Chem. 2020 Jan 23;16:111–24. doi: 10.3762/bjoc.16.13 (PMC7006495; doi:10.3762/bjoc.16.13)
Supplement: File 1 — Additional spectroscopic data, detailed experimental procedures, 1H NMR spectra, and crystallographic data. [file Beilstein_J_Org_Chem-16-111-s001.pdf]

BEILSTEIN JOURNAL OF ORGANIC CHEMISTRY

Supporting Information

for

Reversible photoswitching of the DNA-binding properties of styrylquinolizinium derivatives through photochromic [2 + 2] cycloaddition and cycloreversion

Sarah Kölsch, Heiko Ihmels, Jochen Mattay, Norbert Sewald and Brian O. Patrick

Beilstein J. Org. Chem. **2020,** *16,* 111–124. doi:10.3762/bjoc.16.13

Additional spectroscopic data, detailed experimental procedures, ^{1}H NMR spectra, and crystallographic data

License and Terms: This is a supporting information file under the terms of the Creative Commons Attribution License (https://creativecommons.org/licenses/by/4.0). Please note that the reuse, redistribution and reproduction in particular requires that the authors and source are credited.
The license is subject to the *Beilstein Journal of Organic Chemistry* terms and conditions: (https://www.beilstein-journals.org/bjoc)

Table of Contents

Equipment	S2
Synthesis	S2
Absorption and Emission Spectroscopy	S4
Circular Dichroism (CD) and Flow Linear Dichroism (LD) Spectroscopy	S5
Photometric Monitoring of Photoreactions	S6
Crystallographic Data	S7
^{1}H NMR spectra	S11
References	S15

Equipment

X-ray diffraction analysis: Bruker APEX II area detector diffractometer equipped with a Kryoflex low-temperature device operating at T = 100(2) K. NMR spectroscopy: Bruker AV 400 (^{1}H: 400 MHz; ^{13}C: 100 MHz), Jeol ECZ 500 (^{1}H: 500 MHz; ^{13}C: 125 MHz), Varian 600 ASC (^{1}H: 600 MHz; ^{13}C: 150 MHz). Spectra are determined at ca. 20 °C. Chemical shifts are given in ppm (δ) values and are calibrated relative to the residual solvent peaks. Elemental analyses: HEKAtech *EURO*EA combustion analyser, determined by Mr. Rochus Breuer (Universität Siegen, Organische Chemie I). Mass spectra (ESI): Finnigan LCQ Deca (U = 6 kV; working gas: argon; auxiliary gas: nitrogen; temperature of the capillary: 200 °C). Absorption spectra: Varian Cary 100; fluorescence emission spectra: Varian Cary Eclipse; CD and LD spectra: Chirascan (Applied Photophysics). Melting points: BÜCHI 510 (BÜCHI, Flawil, CH). pH values: pH meter Qph 70, VWR. Photoreactions: Power LED lamp P7QC (LED LENSER) or high-pressure Hg lamp TQ 150 (Heraeus).

Synthesis

General procedure 1 (GP1) for the synthesis of 2-styrylquinolizinium derivatives 3a–d

A solution of 2-methylquinolizinium tetrafluoroborate (**1**, 1.00 mmol) [1], the corresponding aldehyde (1.30 mmol), and piperidine (1.30 mmol) in MeCN (10 mL) was stirred under an argon gas atmosphere at 85 °C for 2–5 h. After cooling the solution to 20 °C, diethyl ether (30 mL) was added, the precipitate was isolated by filtration and washed with diethyl ether (30 mL). The product was crystallized from MeOH/EtOAc.

(*E*)-2-(4'-(Dimethylamino)styryl)quinolizinium tetrafluoroborate (3a) [2]**:** According to GP1, a solution of **1** (231 mg, 1.00 mmol), 4-dimethylaminobenzaldehyde (**2a**, 193 mg, 1.30 mmol), and piperidine (111 mg, 1.30 mmol, 129 µL) in EtOH was stirred for 5 h. The product **3a** was obtained as red prisms (228 g, 639 µmol, 64%); mp >300 °C. – ^{1}H NMR (400 MHz, DMSO-d_6): δ = 3.01 (s, 6 H, CH_3), 6.79 (d, ^{3}J = 9 Hz, 2 H), 7.22 (d, ^{3}J = 16 Hz, 1 H), 7.59 (d, ^{3}J = 9 Hz, 2 H), 7.81–7.86 (m, 2 H), 8.19 (t, ^{3}J = 8 Hz, 1 H), 8.29–8.31 (m, 2 H), 8.35 (s, 1 H, H1), 9.08 (d, ^{3}J = 7 Hz, 1 H), 9.13 (d, ^{3}J = 7 Hz, 1 H).

(*E*)-2-(3',4'-Dimethoxystyryl)quinolizinium tetrafluoroborate (3b): According to the GP1, a solution of **1** (231 mg, 1.00 mmol), 3,4-dimethoxybenzaldehyde (**2b**, 197 mg, 1.30 mmol), and piperidine (111 mg, 1.30 mmol, 129 µL) in EtOH was stirred for 2 h. The product **3b** was obtained as yellow needles (239 mg, 632 µmol, 63%); mp 214–215 °C (decomp.). – ^{1}H NMR (600 MHz, CD_3CN): δ = 3.85 (s, 3 H, OMe), 3.88 (s, 3 H, OMe), 6.97 (d, ^{3}J = 8.0 Hz, 1 H, 5''-H), 7.20 (dd, ^{3}J = 8.0 Hz, 1 H, 6''-H), 7.24 (d, ^{3}J = 16 Hz, 1 H, 1'-H), 7.26 (s, 1 H, 2''-H), 7.65 (d, ^{3}J = 16 Hz, 1 H, 2'-H), 7.75 (dt, ^{3}J = 7 Hz, ^{4}J = 1 Hz, 1 H, 6-H), 8.07 (dd, ^{3}J = 7 Hz, ^{4}J = 2 Hz, 1 H, 3-H), 8.12 (d, ^{3}J = 7 Hz, 1 H, 7-H), 8.18 (s, 1 H, 1-H), 8.20 (d, ^{3}J = 9 Hz, 1 H, 8-H), 8.78–8.81 (m, 2 H, C4, 5-H). – ^{13}C NMR (150 MHz, CD_3CN): δ = 56.5 (2 x OMe), 110.7 (C1'), 112.6 (C5''), 121.6 (C3''), 122.0 (C2''), 123.3 (C1''), 123.6 (C6), 123.7 (C6''), 127.9 (C8), 129.4 (C1), 137.1 (C4 & C5), 137.7 (C7), 140.2 (C2'), 144.5 (C8a), 147.4 (C2), 150.6 (C3''), 152.4 (C4''). – MS (ESI^+): *m/z* (%) = 292 (100) [M^+]. – El. Anal. for $C_{19}H_{18}BF_4NO_2$ (379.16), calcd (%): C 60.19, H 4.79%, N 3.69; found (%): C 60.0, H 4.46, N 3.96.

(*E*)-2-(4'-(Methoxy)styryl)quinolizinium tetrafluoroborate (3c) [2]**:** According to GP1, a solution of **1** (100 mg, 433 µmol), 4-methoxybenzaldehyde (**2c**, 70.7 mg, 519 µmol), and piperidine (51.3 µL, 44.2 mg, 519 µmol) in EtOH was stirred for 2 h. The product **3c** was obtained as yellow needles (130 mg, 342 µmol, 79%). mp 184–186 °C – ^{1}H NMR (400 MHz, DMSO-d_6): δ = 3.83 (s, 3 H, OMe), 7.05–7.06 (m, ^{3}J = 9 Hz, 2 H, 3''-H, 5''-H), 7.41 (d, ^{3}J = 16 Hz, 1 H, 1'-H), 7.73 (d, ^{3}J = 9 Hz, 2 H, 2''-H, 6''-H), 7.91 (d, ^{3}J = 16 Hz, 1 H, 2'-H), 7.95 (dd, ^{3}J = 7, ^{4}J = 2 Hz, 1 H, 3-H), 8.25–8.29 (m, ^{3}J = 7, ^{3}J = 9 Hz, ^{4}J = 1 Hz, 1 H, 7-H), 8.38 (d, ^{3}J = 7 Hz, 1 H, 8-H), 8.41 (d, ^{3}J = 9 Hz, 1 H, 9-H), 8.49 (s, 1 H, 1-H), 9.20 (d, ^{3}J = 7 Hz, 1 H, 4-H), 9.26 (d, ^{3}J = 7 Hz, 1 H, 6-H). – ^{13}C NMR (100 MHz, DMSO-d_6): δ = 55.4 (OMe), 114.5 (C2'', C5''), 120.2 (C9), 121.3 (C1'), 122.2 (C1), 122.4 (C3), 123.4 (C2), 136.4 (C4), 136.7 (C6), 137.4 (C7), 138.4 (C2'), 142.8 (C9a), 142.8 (C1''), 160.8 (C4''). – MS (ESI$^+$): *m/z* (%) = 262 (100) [M$^+$]. – El. Anal. for $C_{18}H_{16}BF_4NO$ (349.14), calcd (%): 61.9; H 4.62, N 4.01; found (%): C 62.09, H 4.59, N 4.39.

(*E*)-2-(4'-(Nitro)styryl)quinolizinium tetrafluoroborate (3d): According to GP1, a solution of **1** (231 mg, 1.00 mmol), 4-nitrobenzaldehyde (**2d**, 197 mg, 1.30 mmol), and piperidine (111 mg, 1.30 mmol, 129 µL) in EtOH was stirred for 4 h. The product **3d** was obtained as yellow needles (289 mg, 764 µmol, 76%), mp 244–246 °C (decomp.). – ^{1}H NMR (600 MHz, CD_3CN): δ = 7.55 (d, ^{3}J = 16 Hz, 1 H, 1'-H), 7.79 (d, ^{3}J = 16 Hz, 1 H, 2'-H), 7.86–7.88 (m, 2 H, 8-H), 7.87 (d, ^{3}J = 9 Hz, 1 H, 2''-H, 6''-H), 8.17 (dd, ^{3}J = 7 Hz, ^{4}J = 2 Hz, 1 H, 3-H), 8.21–8.24 (m, 1 H, 7-H), 8.25 (d, ^{3}J = 7 Hz, 2 H, 3''-H, 5''-H), 8.31 (d, ^{3}J = 9 Hz, 1 H, 9-H), 8.37 (s, 1 H, 1-H), 8.89–8.91 (m, 2 H, 4-H, 6-H). – ^{13}C NMR (150 MHz, CD_3CN): δ = 122.0 (C3''), 124.7 (C8), 125.4 (C1), 125.5 (C3), 128.5 (C9), 128.6 (C1'), 129.8 (C2'', C6''), 137.3 (C2'), 137.7 (C4, C6), 138.5 (C7), 142.9 (C1''), 144.6 (C9a), 145.8 (C2), 149.4 (C4''). – MS (ESI$^+$): *m/z* (%) = 277 (100) [M$^+$]. – El. Anal. for $C_{17}H_{13}BF_4N_2O_2$ (364.11), calc. (%): C 54.73, H 3.78, N 7.51; found (%): C 55.09, H 3.35, N 7.35.

General procedure 2 (GP2) for the photodimerization of 2-styrylquinolizinium derivatives 3b and 3c

A suspension of the styrylquinolizinium derivative (0.50 mmol) in H_2O (150 mL) was irradiated at ca. 450 nm for 6 h with thorough stirring. The product was extracted with $MeNO_2$ (3 × 70 mL), and the organic layers were combined and dried with Na_2SO_4. After filtration, the filtrate was concentrated in vacuum. The product was precipitated from $MeNO_2$ using Et_2O and recrystallized from MeOH or H_2O.

2,2-((*1R*,*2S*,*3R*,*4S*)-2,4-Bis(3'',4''-dimethoxyphenyl)cyclobutane-1,3-diyl)bis(quinolizinium) bis(tetrafluoroborate) (4b): According to GP2, **3b** (150 mg, 396 µmol) was irradiated, and the product **4b** was obtained by recrystallization from H_2O as cubic pale yellow crystals (>99%); mp 167–170 °C. – ^{1}H NMR (600 MHz, DMSO-d_6): *δ* = 3.60 (s, 6 H, OMe'), 3.62 (s, 6 H, OMe), 4.89 (dd, ^{3}J = 10 Hz, ^{3}J = 10 Hz, 2 H, 2'-H), 5.00 (dd, ^{3}J = 10 Hz, ^{3}J = 10 Hz, 2 H, 1'-H), 6.75 (d, ^{3}J = 8 Hz, 2 H, 5''-H), 6.86 (dd, ^{3}J = 8 Hz, ^{4}J = 2 Hz, 2 H, 6''-H), 6.90 (d, ^{4}J = 2 Hz, 2 H, 2''-H), 7.93 (dd, ^{3}J = 7 Hz, ^{4}J = 2 Hz, 2 H, 3-H), 8.01 (dt, ^{3}J = 7 Hz, ^{4}J = 2 Hz, 2 H, 7-H), 8.32–8.34 (m, 2 H, 8-H), 8.40–8.42 (m, 2 H, 9-H), 8.51 (s, 2 H, 1-H), 9.15 (dd, ^{3}J = 7 Hz, 4 H, 4-H, 6-H). – ^{13}C NMR (150 MHz, DMSO-d_6): *δ* = 45.3 (2 x C2'), 46.2 (2 x C1'), 55.3 (2 x OMe'), 55.5 (2 x OMe), 111.4 (2 x C5''), 112.1 (2 x C2''), 119.9 (2 x C6''), 123.2 (2 x C7), 124.2 (2 x C3), 124.6 (2 x C1), 126.3 (2 x C9), 130.5 (2 x C1''), 135.8 (2 x C4), 136.5 (2 x C6), 137.1 (2 x C8), 141.8 (2 x C9a), 147.6 (2 x

C4''),148.4 (2 x C3''), 151.3 (2 x C2). – MS (ESI$^+$): *m/z* (%) = 671 (100) [$M^{2+} BF_4^-$]. – El. Anal. for $C_{38}H_{36}B_2F_8N_2O_4 \times H_2O$ (776.34), calc (%): C 58.79, H 4.93, N 3.61; found: C 58.45, H 4.47, N 3.71.

2,2'-((*1R*,*2R*,*3R*,*4R*)-2,4-Bis(4-methoxyphenyl)cyclobutane-1,3-diyl)bis(quinolizinium) bis(tetrafluoroborate) (4c): According to GP2, **3c** (150 mg, 429 µmol) was irradiated and the product was obtained as pale yellow needles (120 mg, 343 µmol, 80%), mp 211–213 °C. –^{1}H NMR (500 MHz, DMSO-d_6): δ = 3.56 (s, 6 H, OMe), 4.56 (dd, ^3J = 10 Hz, 2 H, 2-H, 4-H), 4.95 (dd, ^3J = 10 Hz, 2 H, 1-H, 3-H), 6.72 (dd, ^3J = 10 Hz, 4 H, 3''-H, 5''-H), 7.20 (dd, ^3J = 6 Hz, 4 H, 2''-H, 6''-H), 7.83 (d, ^3J = 2 Hz, 2 H, 3'-H), 7.91 (d, ^3J = 5 Hz, 2 H, 9'-H), 8.33 (dd, ^3J = 5 Hz, 2 H, 8'-H), 8.41 (dd, ^3J = 5 Hz, 2 H, 7'-H), 8.45 (s, 2 H, 1'-H), 9.00 (d, ^3J = 10 Hz, 2 H, 4'-H), 9.12 (d, ^3J = 10 Hz, 2H, 6'-H). – ^{13}C-NMR (125 MHz, DMSO-d_6): δ = 45.5 (2 C, C2, C4), 46.9 (2 C, C1, C3), 55.3 (2 C, OMe), 114.0 (4 C, C3'', C5''),123.6 (2 C, C9'), 124.0 (2 C, C3'), 126.7 (2 C, C7'), 127.0 (2 C, C8'), 127.4 (2 C, C1'), 130.0 (4 C, C2'', C6''), 136.2 (2 C, C4'), 136.9 (2 C, C6'), 137.0 (2 C, C4''), 142.0 (2 C, C1''), 151.9 (2 C, C2'), 158.5 (2 C, C9a'). – El. Anal. for $C_{36}H_{32}B_2F_8N_2O$ (698.27), calc. (%): C 61.92, H 4.62, N 4.01; found (%): C 62.00, H 4.37, N 4.16.

Absorption and Emission Spectroscopy

Preparation of solutions for spectrometric analyses

The ct DNA was dissolved in BPE buffer and stored at 4 °C for at least 24 h and filtered. The concentration (in base pairs, bp) was determined photometrically (λ_{max} = 260 nm, ε = 12824 $cm^{-1} \cdot M^{-1}$).

BPE buffer was prepared from biochemistry-grade chemicals (Fluka BioChemika Ultra) and e-pure water: c = 6.0 mM Na_2HPO_4, c = 2.0 mM NaH_2PO_4, and c = 1.0 mM Na_2EDTA; c = 16 mM total Na^+. Prior to use, the buffer solutions were filtered through a PVDF membrane filter (pore size 0.45 µm).

Stock solutions were prepared by dissolving **3a**–**d** in acetonitrile or water (**4b**) to give a concentration of 1.0 mM. The solutions were stored at 4 °C. All measurements were performed in thermostated quartz cuvettes with a path length of d = 1 cm at 20 °C, if not stated otherwise.

The absorption and emission spectra were recorded with a scan rate of 120 nm/min. The detection wavelength range of the absorption spectra was 200–600 nm with slit widths of 2 nm, and the recorded range of the emission spectra was 400–800 nm. Every experiment was performed at least two times. The fluorescence spectra were recorded with a detection voltage of U = 700 V and an excitation and emission slit of 5 nm, if not stated otherwise. The excitation wavelength for **3a** was at the isosbestic point at λ = 370 nm, the excitation wavelength of **3b** was λ = 410 nm and **3c** λ = 404 nm.

Aliquots of the stock solutions of compounds **3a**–**d** were pipetted in graduated flasks. The solvent was evaporated and the residue was dissolved in BPE buffer (2.0 mL) to obtain the required concentration. The titrant solution with ct DNA (c = 1.0–2.5 mM) contained the same concentration of ligand as the analyte solution. The analytes (1.0–2.0 mL) were pipetted into the quartz cuvettes and titrated with the titrant solutions in 0.25–20 equivalent intervals until no changes in absorption or fluorescence intensity were observed.

The binding constants were determined according to published procedures [3] from the spectrophotometric titrations (Figure S1) and fitting of the experimental binding to a theoretical

model considering noncompetitive binding [3], respectively (Figure S1A–C). Standard deviations (SD) of K_b values were calculated from Equation 1.

$$SD\ (K_b) = ((SD\ of\ A/A)/A)/c_{lig} \qquad (1)$$

SD = standard deviation

A = value of the absorption

SD of A is used as provided by the Origin 7.5 software.

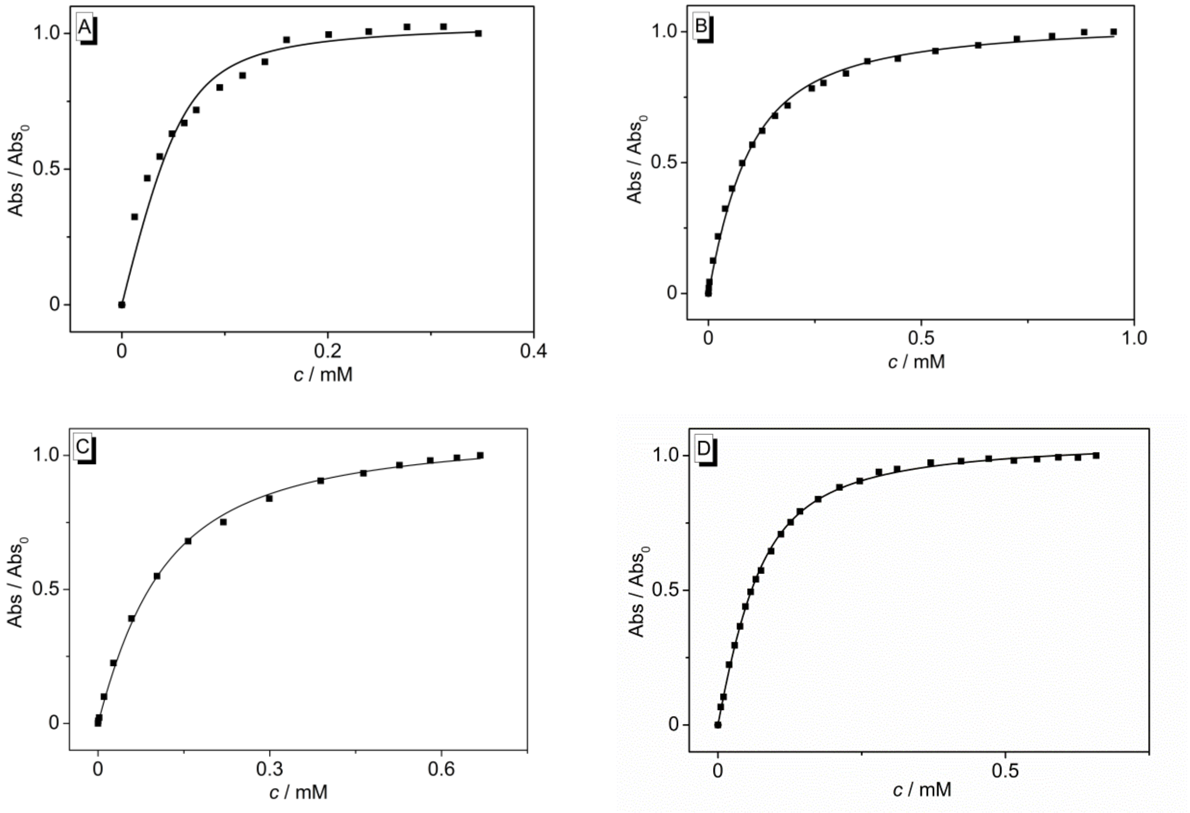

Figure S1: Plots of normalized absorption vs ct DNA concentration and the corresponding fitting of the experimental data to the theoretical model (A: **3a**, B: **3b**, C: **3c**; D: **3d**).

Fluorescence quantum yields of derivatives **3a**–**d** in acetonitrile solutions were determined according to published procedures [4].

Circular Dichroism (CD) and Flow Linear Dichroism (LD) Spectroscopy

The analyte solutions were prepared by pipetting different aliquots of the stock solution of the ligands into Eppendorf vials and then the solvent was removed. Buffer solution and a solution of ct DNA in BPE buffer were added to adjust the concentration and the ligand–DNA values. The

spectra were recorded at λ = 230−600 nm with a band width of 1 nm and a scan rate of 1 nm·s^{-1} with a time per point of 0.5 s. The samples were recorded three times and averaged. The spectra were smoothed with the Savitzky–Golay method and implemented in the Chirascan software, with a polynomial order of 5.

Photometric Monitoring of Photoreactions

Aliquots of the stock solutions of compounds **3a**–**d** were pipetted in Eppendorf vials, the solvent was evaporated, and the residue was dissolved in MeCN or H_2O to obtain a final concentration of *c* = 20–25 µM. The samples were irradiated at ca. 530 nm (**3a**) and ca. 450 nm (**3b**, **3c**) with an LED lamp or a high-pressure Hg lamp with cut-off filter (>395 nm, **3d**). The reactions were monitored photometrically with a scan rate of 120 nm/min in a range of 200–600 nm (Figure S2).

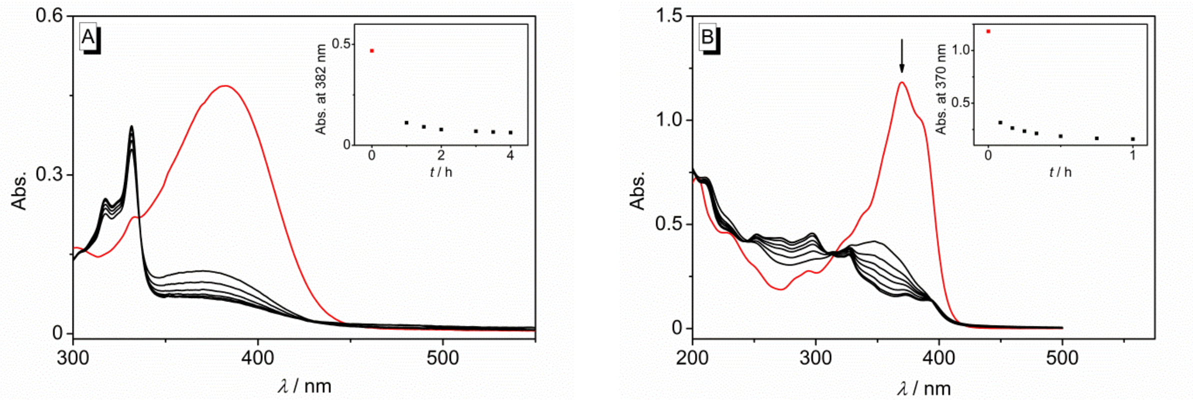

Figure S2: Photometric analysis of the photocycloaddition of **3c** (A, 20 µM) and **3d** (B, 25 µM) in H_2O.

Crystallographic Data

Photodimer 4b

Data were measured using ϕ and ω scans of 0.5° per frame for 90 s with MoK_α radiation (microfocus sealed X-ray tube, 50 kV, 0.99 mA). The total number of runs and images was based on the strategy calculation from the program APEX3. The maximum resolution that was achieved was Θ = 22.469° (0.93 Å).

The unit cell was refined using SAINT (Bruker, V8.38A, after 2013) on 9305 reflections, 54% of the observed reflections. Data reduction, scaling and absorption corrections were performed using SAINT (Bruker, V8.38A, after 2013). The final completeness was 99.70% out to 22.469° in Θ.

A multiscan absorption correction was performed using SADABS-2016/2 (Bruker, 2016/2) and used for absorption correction. wR_2(int) was 0.0879 before and 0.0599 after correction. The ratio of minimum to maximum transmission was 0.7849. The $\lambda/2$ correction factor is not present. The absorption coefficient μ of this material was 0.114 mm^{-1} at this wavelength (λ = 0.71073 Å), and the minimum and maximum transmissions were 0.773 and 0.985.

The structure was solved in space group $P2_1/n$ with the XT [5] structure solution program using the intrinsic phasing solution method and by using Olex2 [5] as the graphical interface.

The material crystallized with significant disorder. The orientation of one quinolizinium unit was found in two different orientations. Each fragment was refined using similarity restraints on bond lengths and angles. In addition, one BF_4 anion was modelled in three orientations, with restraints on all B–F bond lengths. Finally, the material crystallized with disordered solvent in the lattice. This solvent could not be modeled, therefore, the PLATON/SQUEEZE program [6] was employed to generate a 'solvent-free' data set. All nonhydrogen atoms were refined anisotropically. Hydrogen atom positions were calculated geometrically and refined using the riding model.

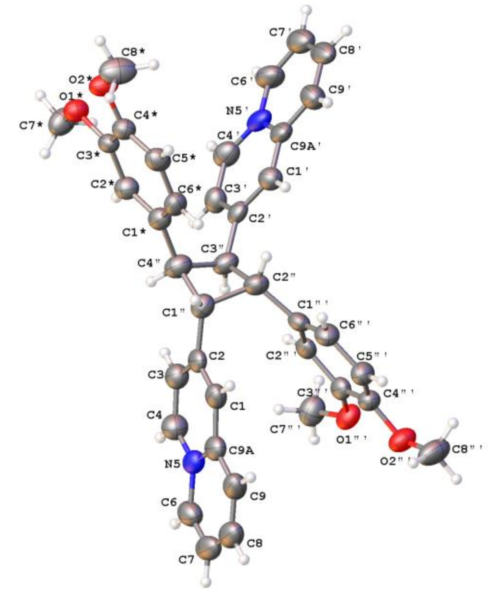

Figure S3: ORTEP image of the major disordered fragment of **4b**. All ellipsoids are drawn at 50% probability. The BF_4 anions have been removed for clarity.

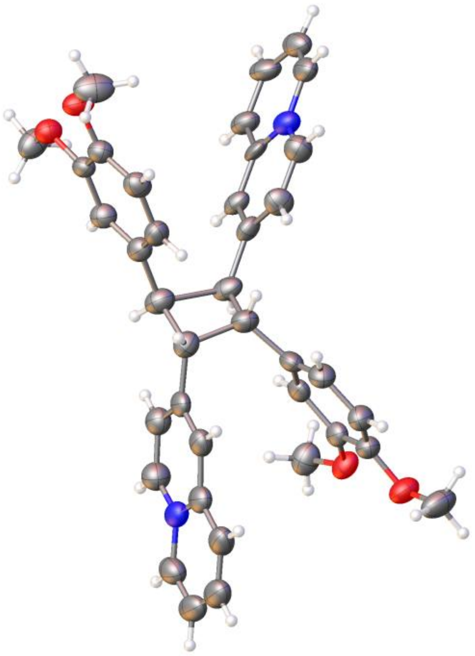

Figure S4: ORTEP image of the minor disordered fragment of **4b**. All ellipsoids are drawn at 50% probability. The BF_4 anions have been removed for clarity.

structure quality indicators:

reflections:	d min (Mo) 0.93	I/σ 20.9	Rint 4.31%	complete 100%
refinement:	Shift 0.000	Max Peak 0.8	Min Peak -0.3	GooF 1.106

Photodimer 4c

Single colorless irregular shaped crystals of **4c** were recrystallized from water by slow evaporation. The structure was solved with the XT structure solution program using the intrinsic phasing solution method and by using Olex2 [5] as the graphical interface. The model was refined with version 2018/3 of XL using least-squares minimization.

Data were measured using ϕ and ω scans of 0.5° per frame for 30 s using MoK_{α} radiation (microfocus sealed X-ray tube, 50 kV, 0.99 mA). The total number of runs and images was based on the strategy calculation from the program APEX3. The maximum resolution that was achieved was Θ = 26.468° (0.80 Å).

The diffraction pattern was indexed and the unit cell was refined using SAINT (Bruker, V8.38A, after 2013) on 7034 reflections, 51% of the observed reflections. Data reduction, scaling and absorption corrections were performed using SAINT (Bruker, V8.38A, after 2013). The final completeness was 100% out to 26.468° in Θ.

A multiscan absorption correction was performed using SADABS-2016/2 (Bruker, 2016/2) and used for absorption correction. wR_2(int) was 0.0913 before and 0.0629 after correction. The ratio of minimum to maximum transmission was 0.8424. The $\lambda/2$ correction factor is not present. The absorption coefficient μ of this material was 0.119 mm^{-1} at this wavelength (λ = 0.711 Å), and the minimum and maximum transmissions were 0.628 and 0.745.

The structure was solved and the space group *P*-1 (# 2) determined by the XT [5] structure solution program using intrinsic phasing and refined by least squares using version 2018/3 of XL [5]. The material crystallized with a small fraction (ca. 7%) of near whole molecule disorder, with the two fragments related by a 180° rotation and oriented such that the methoxyphenyl and quinolizinium moieties were nearly overlapping. Modeling of the minor fraction required the use of restraints and constraints to maintain reasonable geometries. All nonhydrogen atoms were refined anisotropically. Hydrogen atom positions were calculated geometrically and refined using the riding model. The value of Z' was 0.5. This means that only half of the formula unit was present in the asymmetric unit, with the other half consisting of symmetry-equivalent atoms.

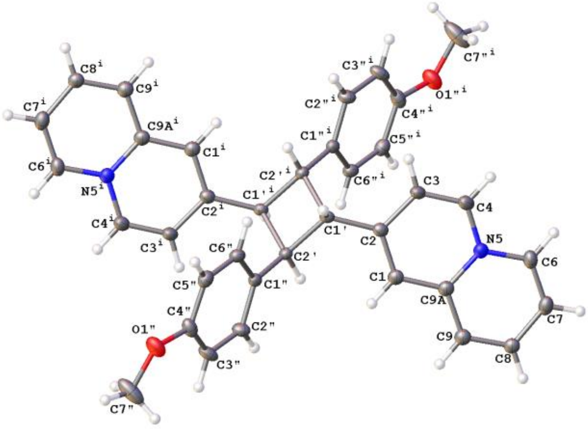

Figure S5: ORTEP image of the major disordered fragment of **4c**. All ellipsoids are drawn at 50% probability. The BF_4 anions have been removed for clarity.

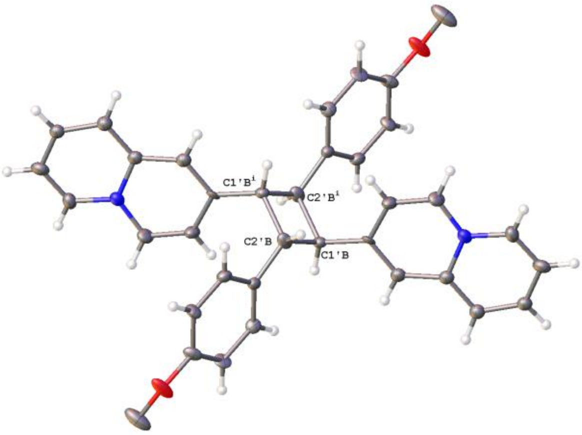

Figure S6: ORTEP image of the minor disordered component of **4c**. The BF_4 anions have been removed for clarity.

structure quality indicators

reflections:	d min (Mo) 0.80	I/σ 24.7	Rint 4.69%	complete 100%
refinement:	Shift 0.000	Max Peak 0.3	Min Peak -0.4	GooF 1.040

Table S1: Crystal data and structure refinement details of **4b** and **4c**.[a]

compound	4b	4c
formula	$C_{38}H_{36}B_2F_8N_2O_4$	$C_{36}H_{32}B_2F_8N_2O_2$
$D_{calc.}$/g·cm^{-3}	1.362	1.430
μ/mm^{-1}	0.114	0.119
formula Wweight	761.13	698.25
color	colorless	colorless
shape	prism	irregular
size/mm^3	0.25·0.15·0.13	0.24·0.23·0.08
T/K	100(2)	100(2)
crystal system	monoclinic	triclinic
space group	$P2_1/n$	P-1
a/Å	13.339(2)	9.062(3)
b/Å	15.093(2)	9.986(3)
c/Å	18.815(3)	10.433(3)
α/°	90	91.011(9)
β/°	101.478(4)	114.226(9)
γ/°	90	107.399(10)
V/Å^3	3712.2(9)	810.8(4)
Z	4	1
Z'	1	0.5
wavelength/Å	0.71073	0.71073
radiation type	MoK_α	MoK_α
Θ_{min}/°	1.721	2.166
Θ_{ma}x/°	22.469	26.468
measured refl's	17353	13777
ind't refl's	4833	3335
refl's with I > 2(I)	3516	2635
R_{int}	0.0431	0.0469
parameters	651	321
restraints	1692	1060
largest Peak	0.824	0.326
deepest Hole	−0.333	−0.400
GooF	1.106	1.040
wR_2 (all data)	0.3065	0.1357
wR_2	0.2887	0.1257
R_1 (all data)	0.1460	0.0635
R_1	0.1178	0.0481

[a]CCDC deposition numbers 1963881 (**4b**) and 1963882 (**4c**) contain the supplementary crystallographic data for these compounds. These data can be obtained at The Cambridge Crystallographic Data Centre at https://www.ccdc.cam.ac.uk.

^{1}H NMR spectra

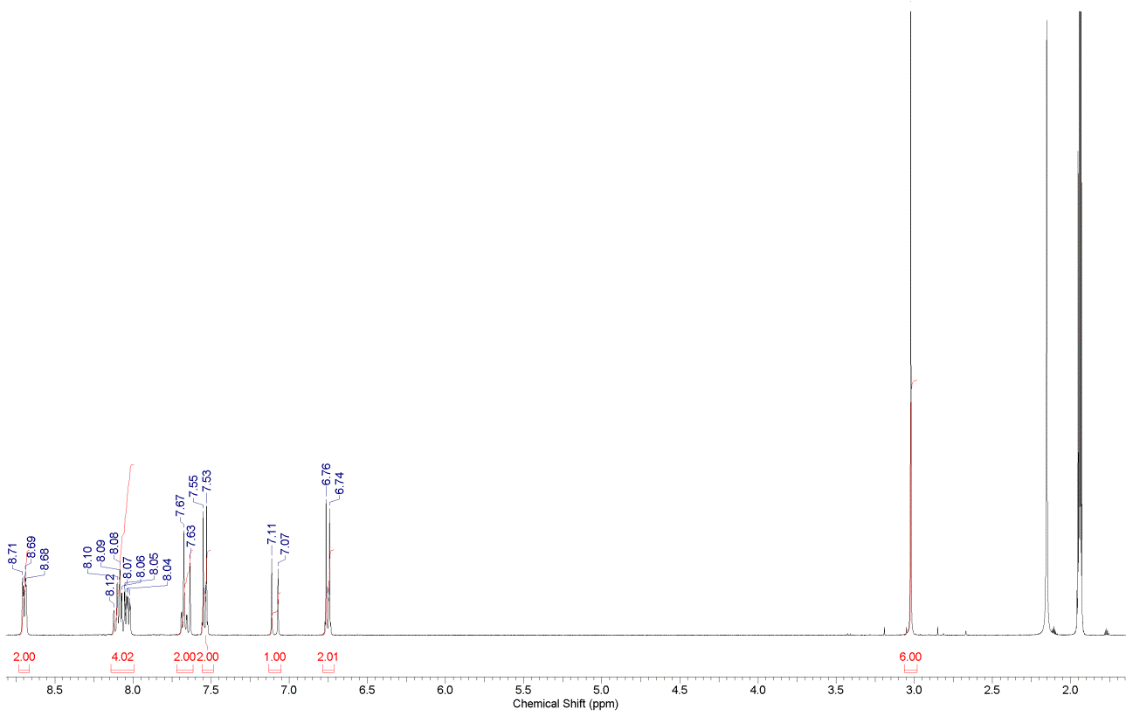

Figure S7: ^{1}H NMR spectrum (400 MHz) of **3a** in CD_3CN.

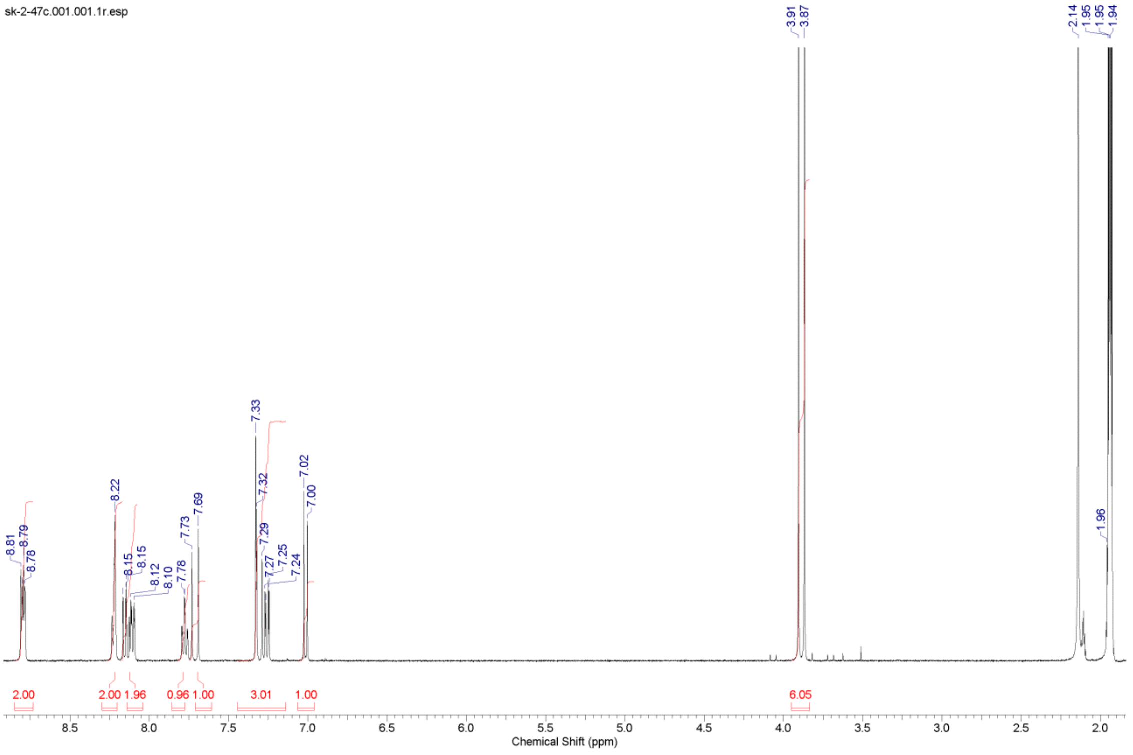

Figure S8: ^{1}H NMR spectrum (600 MHz) of **3b** in CD_3CN.

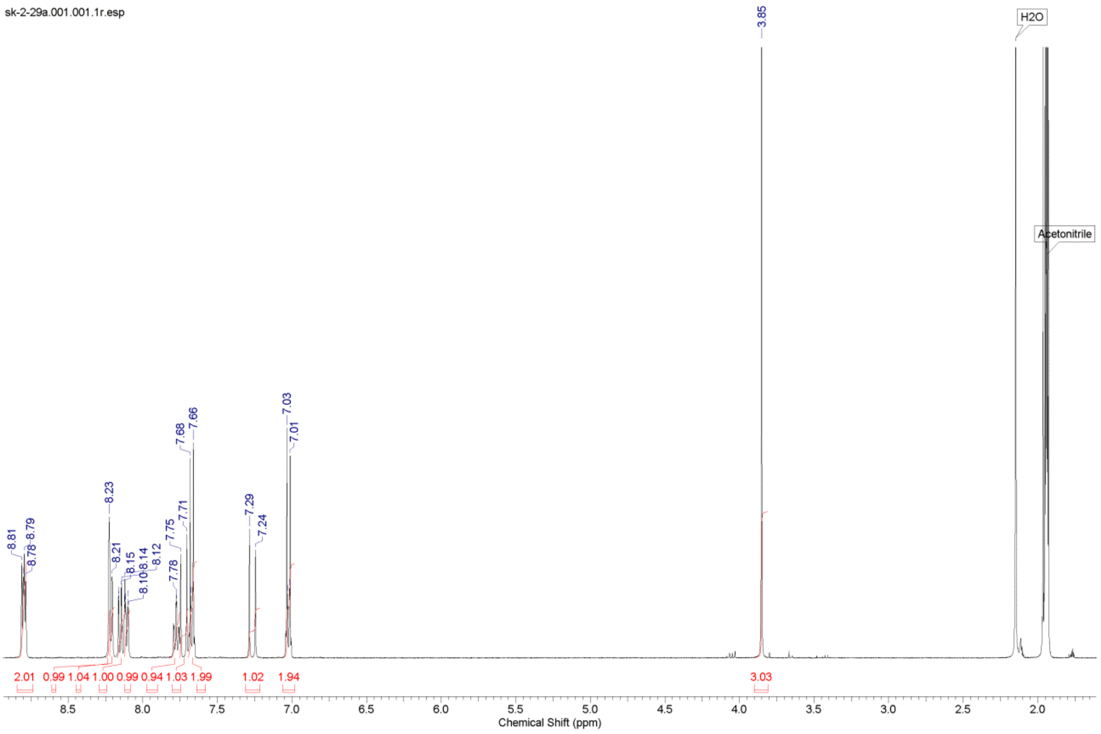

Figure S9: ^{1}H NMR spectrum (400 MHz) of **3c** in CD_3CN.

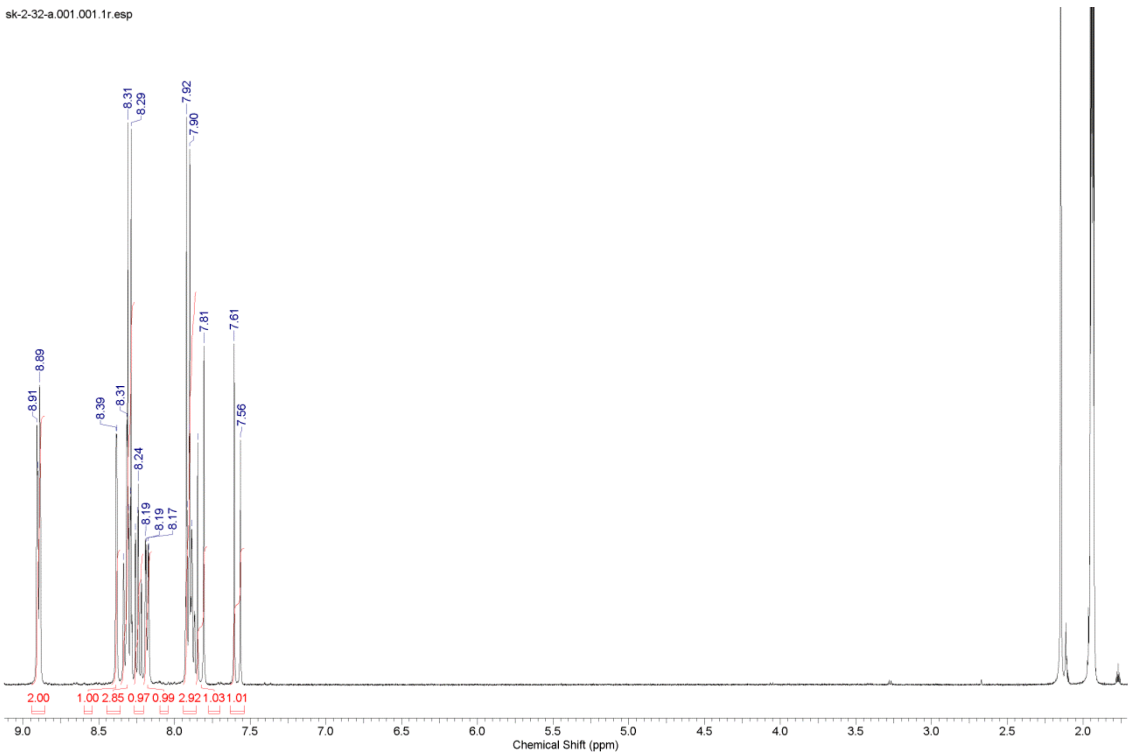

Figure S10: ^{1}H NMR spectrum (600 MHz) of **3d** in CD_3CN.

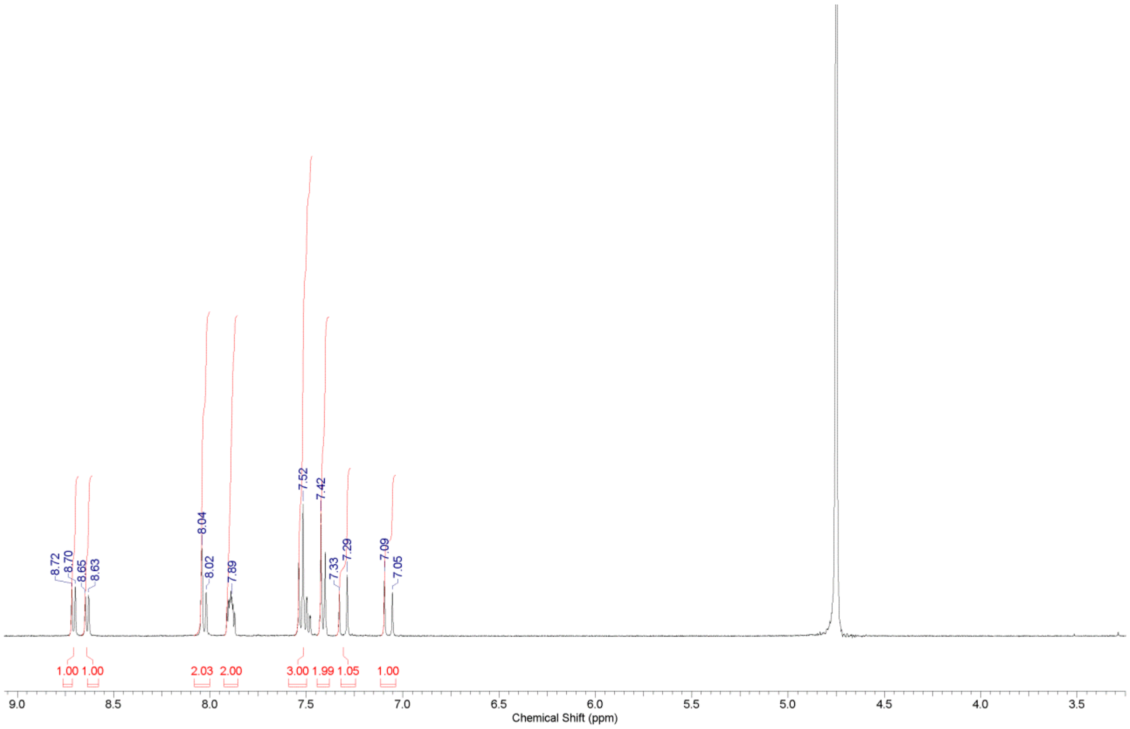

Figure S11: ^{1}H NMR spectrum (400 MHz) in D_2O after irradiation of **3a** at 530 nm for 2 h.

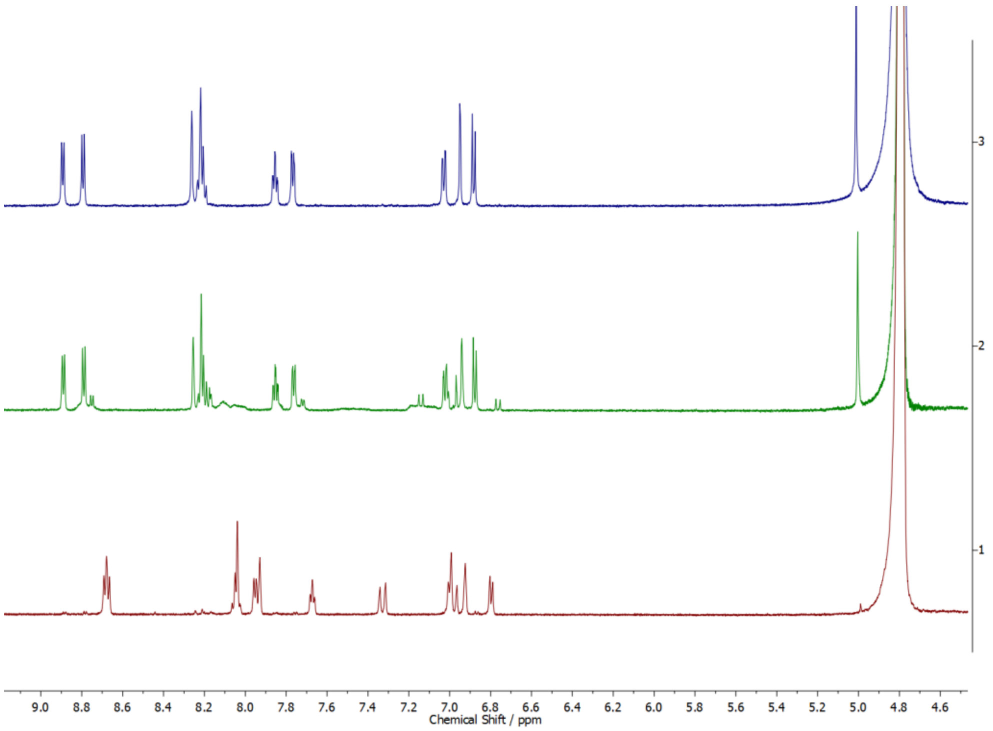

Figure S12: Irradiation of **3b** at ca. 450 nm (LED) in D_2O followed by ^{1}H NMR spectroscopy (600 MHz, bottom: 0 min, middle: 1 min, top: 3 min).

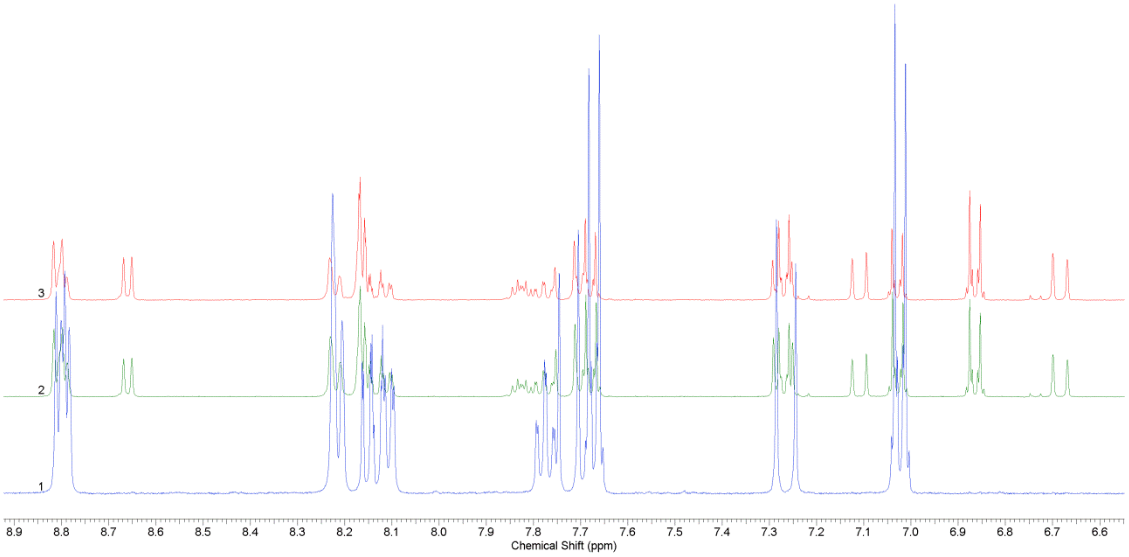

Figure S13: Irradiation of **3c** at ca. 530 nm (LED) in CD_3CN followed by ^1H NMR spectroscopy (400 MHz, 1: 0 h, 2: 2 h, 3: 6 h).

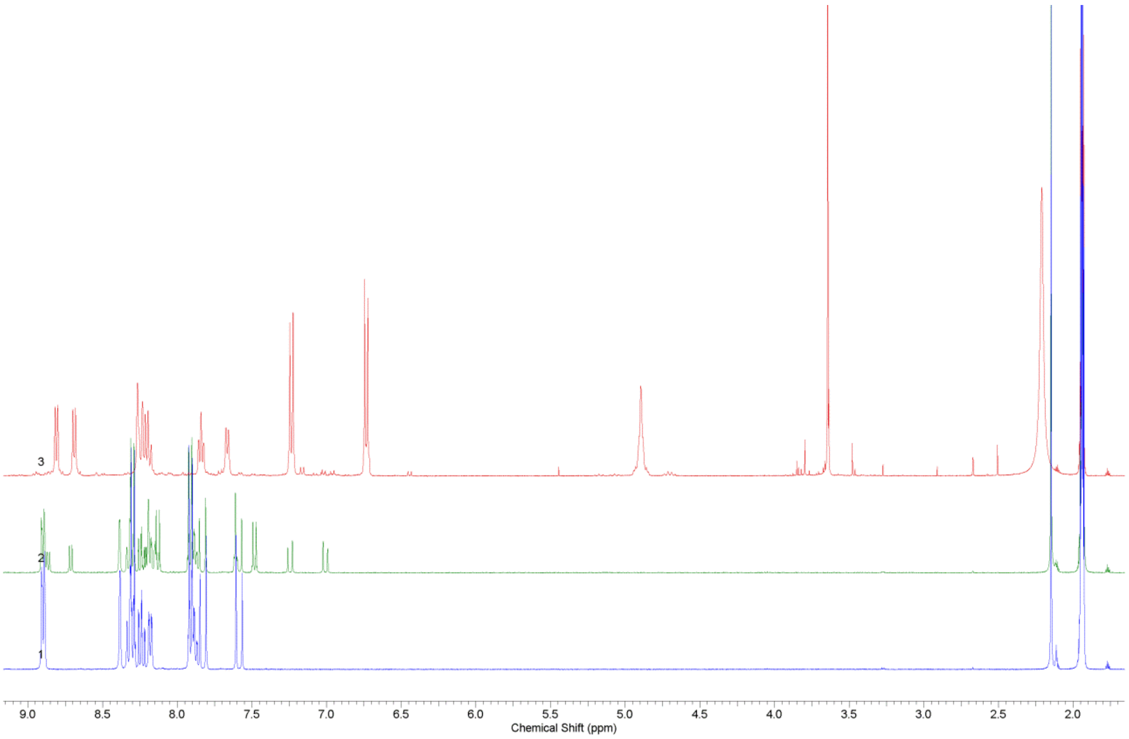

Figure S14: Irradiation of **3d** at 365 nm (Hg high-pressure lamp) in CD_3CN followed by ^1H NMR spectroscopy (400 MHz, 1: 0 h, 2: 2 h, 3: 8 h).

References

[1] a) Beumel, O. F.; Novis Smith, W.; Rybalka, B. *Synthesis* **1974**, *1*, 43–45. b) Richards, A.; Stevens, T. S. *J. Chem. Soc.* **1958**, 3067–3073.

[2] Marcelo, G.; Pinto, S.; Caneque, T.; Mariz, I. F. A.; Cuadro, A. M.; Vaquero, J. J.; Martinho, J. M. G.; Macoas, E. M. S. *J. Phys. Chem. A* **2015**, *119*, 2351–2362.

[3] Stootman, F. H.; Fisher, D. M.; Rodger, A. Aldrich-Wright, J. R. *Analyst* **2006**, *131*, 1145–1151.

[4] a) Pithan, P. M.; Decker, D.; Druzhinin, S. I.; Ihmels, H.; Schönherr, H.; Voß, Y. *RSC Advances*, **2017**, *7*, 10660–10667. b) Das, A. K.; Druzhinin, S. I.; Ihmels, H.; Müller, M.; Schönherr, H. *Chem. Eur. J.* **2019**, *25*, 12703–12707.

[5] Dolomanov, O. V.; Bourhis, L. J.; Gildea, R. J.; Howard, J. A. K.; Puschmann, H. *J. Appl. Cryst.* **2009**, *42*, 339–341.

[6] Spek, A. L. *Acta Cryst.* **2015**, *C71*, 9–18.
